# Why the HIV Reservoir Never Runs Dry: Clonal Expansion and the Characteristics of HIV-Infected Cells Challenge Strategies to Cure and Control HIV Infection

**DOI:** 10.3390/v13122512

**Published:** 2021-12-14

**Authors:** Chuen-Yen Lau, Matthew A. Adan, Frank Maldarelli

**Affiliations:** 1 HIV Dynamics and Replication Program, NCI, NIH, Bethesda, MD 20892, USA; chuen-yen.lau@nih.gov (C.-Y.L.); Matthew.adan@nih.gov (M.A.A.); 2 Vagelos College of Physicians & Surgeons, Columbia University, New York, NY 10032, USA

**Keywords:** HIV, clonal expansion, reservoir, persistence, cure

## Abstract

Antiretroviral therapy (ART) effectively reduces cycles of viral replication but does not target proviral populations in cells that persist for prolonged periods and that can undergo clonal expansion. Consequently, chronic human immunodeficiency virus (HIV) infection is sustained during ART by a reservoir of long-lived latently infected cells and their progeny. This proviral landscape undergoes change over time on ART. One of the forces driving change in the landscape is the clonal expansion of infected CD4 T cells, which presents a key obstacle to HIV eradication. Potential mechanisms of clonal expansion include general immune activation, antigenic stimulation, homeostatic proliferation, and provirus-driven clonal expansion, each of which likely contributes in varying, and largely unmeasured, amounts to maintaining the reservoir. The role of clinical events, such as infections or neoplasms, in driving these mechanisms remains uncertain, but characterizing these forces may shed light on approaches to effectively eradicate HIV. A limited number of individuals have been cured of HIV infection in the setting of bone marrow transplant; information from these and other studies may identify the means to eradicate or control the virus without ART. In this review, we describe the mechanisms of HIV-1 persistence and clonal expansion, along with the attempts to modify these factors as part of reservoir reduction and cure strategies.

## 1. Introduction

Antiretroviral therapy (ART) successfully halts the replication of the HIV virus and prevents the infection of new cells in blood and tissue [1,2,3]. While ART reduces the morbidity and mortality associated with a decline in CD4 T cells and the progression to AIDS, ART does not result in a cure [4,5,6], and HIV persistence represents the most important obstacle preventing the eradication of virus infection or viral control without ART. Despite long-term suppressive therapy, very low levels of viremia persist in those on ART, in the range of 1–2 copies/mL of plasma, and the HIV viral load (plasma HIV ribonucleic acid (RNA) per mL of blood) typically returns with the cessation of treatment. Rebound viremia arises from an HIV reservoir, defined here as a population of cells infected with replication-competent HIV virus. The replication-competent reservoir is only a small proportion (c. 1–2%) of the population of HIV-infected cells, the remainder of which are infected with replication-incompetent HIV. As recently demonstrated, however, HIV-incompetent proviruses may produce HIV RNA and protein, which may be recognized by host immune cells, and may contribute to immune activation, which has been correlated with morbidity and mortality during ART [7,8,9]. Consequently, both replication-competent and -incompetent proviruses may contribute to HIV pathogenesis during ART. The characterization of different HIV-infected cell populations, including latently infected cells, will provide insight into HIV persistence and cure strategies.

Infected cell populations can be characterized in diverse ways, including by constructing phylogenies, determining the integration site and the replication competence of the proviruses, and measuring the levels of virion production in plasma. An examination of phylogenetic relationships on the basis of proviral sequences would reveal the diversity of the different populations. Identifying the integration site of proviruses will shed light on the presence and size of the clonal populations of infected cells. Determining the replication competence of proviruses identifies the proportion of infectious proviruses that may rebound if ART is interrupted and that require eradication for cure. Quantifying viremia represents an important reservoir characteristic. Although viremia is typically low (1–2 copies/mL plasma) during ART, the substantial overall blood volume (c. 5 L) and the relatively short half-life of virions in blood (15 min–3 h) suggest that large numbers of cells are producing virions to maintain this level of viremia. Although not all virions present in plasma are replication-competent, they do reflect a population capable of virion production and can present a source of broad antigenic stimulation. In vivo, infected cells persist within a number of anatomic locations, where they may continue to have a role in ongoing disease pathogenesis. The role of these locations in HIV persistence remains uncertain, but an understanding is essential in order to identify useful strategies that ensure HIV eradication from these compartments.

The ultimate goal of understanding HIV persistence is the design of effective and scalable strategies to either eradicate HIV (sterilizing cure) or control HIV infection, without the requirement of ART (functional cure). Several approaches, including “shock-and-kill”, “block-and-lock”, ART intensification, and activated CD8 T-cell infusions have been employed in an attempt to reduce reservoirs [10,11,12,13,14]. Thus far, these approaches have had no detectable long-term impact on the replication-competent reservoir. Only bone marrow transplantation for life-threatening neoplastic disease has led to examples of HIV cure with a complete eradication of the virus. There has been speculation that some individuals with a natural control of HIV infection with viral-RNA-suppressed <50 c/mL plasma (denoted “elite” controllers) may be able to achieve functional cure, in which HIV is controlled without the requirement for ART [15,16].

We herein review mechanisms of HIV-1 persistence and clonal expansion, along with attempts to modify these factors, as part of reservoir reduction and cure strategies. For a review of HIV clonal expansion and reservoir measurement assays, see [17,18,19,20]. Others have reviewed HIV integration and integration assays [21,22,23,24]. Animal models of HIV infection provide critical information regarding HIV persistence but are beyond the scope of this review and are reviewed elsewhere [25,26,27,28,29]. We will discuss the role of antivirals as they relates to the lifecycle and persistence; the underlying molecular mechanisms of action and resistance will not be addressed. Several recent reviews address this topic [30,31,32], and the Stanford University HIV Drug Resistance Database (https://hivdb.stanford.edu/ accessed on 29 November 2021) maintains a detailed discussion of the resistance to each antiretroviral [33].

### 1.1. HIV Replication and Establishing the Proviral State

Understanding the HIV lifecycle is critical for understanding the mechanisms of persistence, the role of the clonal expansion of HIV-infected cells in maintaining HIV-infected cell populations, and approaches to HIV cure. While HIV commonly infects CD4 T cells, the virus has been demonstrated in other cell types, such as macrophages, in diverse anatomic locations. Infection begins with the binding of envelope gp120 to the CD4 T-cell receptor (TCR), and then also to a coreceptor, most commonly either the CCR5 or CXCR4 seven transmembrane G-protein coupled chemokine receptor. When both the CD4 and the chemokine coreceptor are bound, the membrane proximal region undergoes a conformational change and a fusion of the virion and the CD4 T-cell membranes occur. The viral core is released into the host cell cytoplasm; subsequently, the viral genome requires reverse transcription and integration into the host genome.

Reverse transcription is an essential step in retroviral replication. In this process, RNA is first transcribed to an RNA-deoxyribonucleic acid (DNA) hybrid, after which the RNA strand is removed, and the second strand of DNA is synthesized. Because of this, reverse transcriptase is both an RNA-dependent DNA polymerase and a DNA-dependent DNA polymerase, and it has RNase H activity. Reverse transcription is highly error-prone; early studies reveal that a single viral replication cycle induced the forward mutation rate of c. 3.4 × 10^−5^ per site/replication cycle, or approximately one new mutation for each complete genome replication [34,35]. Recombination during reverse transcription is common and, indeed, essential for HIV replication, [36] and 6–7 strand transfers occur during each reverse transcription event (reviewed in Hu and Hughes ([37]). From 15–20% of the substitution mutations that occurred during reverse transcription were associated with recombination events [38]. Recombination events may not be completely random. It has been suggested that they occur at different rates in “hot” and “cold” areas, on the basis of studies with *gag* and *pol* [39], though in vitro findings may not completely recapitulate the patterns of mutagenesis or recombination that occur in in vivo [40]. The combination of rapid replication, a high mutation rate, and frequent recombination result in a population of viruses with relatively high genetic diversity; it has been estimated that mutations at every site in the HIV genome may be generated daily [41,42]. Defects that render proviruses noninfectious include deletions and mutations accumulate in vivo, including G-to-A mutations due to the apolipoprotein B messenger RNA-editing enzyme, catalytic polypeptide (APOBEC)-mediated cytidine deamination [43]. However, regardless of mutation or deletion, essentially all products of reverse transcription are substrates for integration. As a result, comparatively few infected cells contain replication-competent proviruses and many infected cells contain defective proviruses, which has profound consequences for the establishment of infected cell populations and their persistence once ART is introduced.

Until recently, it was generally assumed that the uncoating of the viral core took place in the cytoplasm followed by reverse transcription. Although defective particles may uncoat in the cytoplasm, elegant studies by Pathak and coworkers recently demonstrated that effective reverse transcription leading to integration actually initiates in the nucleus, or at the nuclear membrane and is completed in the nucleus. Uncoating follows reverse transcription and also takes place in the nucleus [44]. This new information on the location of reverse transcription and uncoating has critical consequences for understanding the action of antiretroviral agents and the establishment of the HIV reservoir. For instance, by completing reverse transcription in the nucleus, HIV avoids activating the cytoplasmic cyclic guanosine monophosphate-adenosine monophosphate synthase (cGAS)-directed innate immune-sensing of DNA. If the core undergoes uncoating before entering the nucleus, infection may be aborted, and innate immunity may be stimulated. Once in the nucleus, reverse transcription is completed; surprisingly, optimum reverse transcription requires core stability. Uncoating follows quickly after reverse transcription is completed in preparation for integration [45,46].

**Figure 1:**
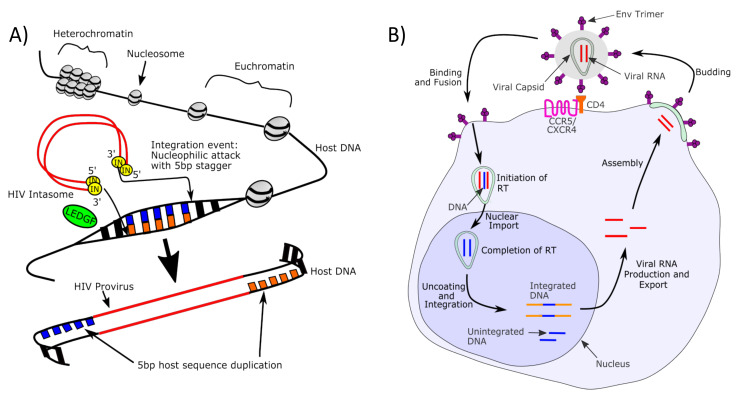
(**A**) Mechanism of HIV integration. HIV proviruses integrate into host DNA via a nucleophilic attack, staggered by 5 host base pairs. This staggering of base pairs allows for integration site analysis to determine true clonal expansion from the results of polymerase chain reaction (PCR) amplification. HIV integration favors transcriptionally active regions of the chromosome and, therefore, typically does not take place within tightly bound heterochromatin. LEDGF/p75 is a transcriptional activator that guides integration towards transcriptionally active regions and AT-rich regions. A simplified representation of the HIV intasome is depicted. It is likely comprised of a multimer of integrase molecules. Bp: base pair; IN: integrase tetramer. Adapted from [64]. (**B**) Phases of the productive HIV replication cycle. HIV proviruses also replicate through clonal expansion, not depicted here. RT: reverse transcription.

HIV integration into the host genome is central to HIV replication and proviral persistence. As shown in Figure 1A, integration is mediated by a large complex “intasome” that contains integrase, as well as cellular cofactors, such as lens epithelial-derived growth factor/protein 75 (LEDGF/p75). LEDGF/p75 and cleavage and polyadenylation specificity factor subunit 6 (CPSF6) are host proteins involved in integration site selection [47,48]. As part of the intasome, HIV has co-opted LEDGF/p75 to function as a chaperone, targeting the HIV DNA to the regions of the genome undergoing active transcription [47,49]. Once the intasome comes in close proximity to the host genome, integration can take place. The integrase, assembled as multimers at the ends of the newly reverse-transcribed DNA, digest two nucleotides from the 3′ends of HIV DNA, creating a two-base-pair staggered end. Next, the integrase introduces two endonucleolytic cuts in the host DNA, creating a two-base-pair overhang. At the host site, integrase catalyzes a concerted transesterification reaction, which first introduces two endonucleolytic cuts (one on each strand) of the host genome, separated by five base pairs, and introduces the HIV sequence at the site of the cut. Once strand transfer has taken place, host DNA polymerases fill in the unpaired nucleotides generated by the staggered cut, and host ligases complete the repair. As a result, there is a five-base-pair duplication of the host sequence at each end of the provirus integration site (Figure 1A, for review see [50]).

Several characteristics of integration events have important consequences for understanding HIV persistence and the establishment of long-term HIV populations:

1. Sites of integration are widely distributed in the genome. Integrations generally take place at the 5′AAT, 5′AAA, and 5′ TAA sequences. There are, in general, no other sequence specificities and, in general, no specific “hot spots” for integration;
2. Integration preferences exist, generally on the basis of the LEDGF/p75 chaperone preference for regions of active transcription. Proviruses are more commonly found in introns over exons because of the greater relative size of introns in the human genome. Importantly, promoter regions are largely excluded from LEDGF/p75-directed integration. As such, the HIV promoter is rarely present within a host gene promoter, which makes the strong transcriptional effects of the HIV long terminal repeat (LTR) on the host genes, such as those which drive oncogene expression in neoplasms, less likely [51].
3. The integration event is independent with respect to the orientation of the transcription of the host gene. Proviruses may be introduced with viral and host transcriptions in the same orientation, or in an opposite orientation.
4. Integration is independent of the size of the provirus and intact and deleted HIV DNA forms are readily integrated.

As a result of these characteristics, which can be demonstrated in in vitro systems [52], infected cells entering the long-term HIV population generally have proviruses that are widely distributed in the transcriptionally active areas of the genome. As a group, the proviruses are present in an orientation that is independent of the host gene transcription. As described below, from this initial state, infected cells will undergo both positive and negative selection, both prior to and following the initiation of ART. In some instances, HIV DNA fails to integrate into the host genome, but may undergo end-to-end autointegration, resulting in circular forms of HIV DNA containing one or two LTR copies. Autointegration is mediated by host ligases, not by HIV integrase. Circular forms of HIV generally do not express HIV RNA, likely because of the high histone loading [53], and circular HIV DNA does not undergo replication when the host cell divides. Thus, 1- and 2-LTR circles are gradually diluted by cell replication. Nevertheless, circular HIV DNA may persist for prolonged periods, similar to other episomal DNA forms.

Once integration is complete, active replication proceeds with transcription to mRNA, which undergoes alternative splicing and is exported out of the nucleus [54,55,56]. Late steps in HIV replication include the transport of viral structural and envelope proteins, the assembly of virions, the budding of virions from the cell surface, and the maturation of virions into infectious particles through the action of protease (see [57] for review). It has been estimated that an activated and productively infected cell can release several thousand viral particles per day [58]. A schematic of the HIV replication cycle is shown in Figure 1B.

In general, HIV infection takes place in activated cells. A small fraction of cells does not remain activated after integration and does not produce HIV RNA or undergo elimination. Cells that have reverted to a quiescent state may persist and seed a long-lived reservoir [59,60,61,62]. Recent studies have suggested that quiescent and naïve cells themselves can become infected with HIV and can directly seed the HIV reservoir [63]. Although an infection of quiescent cells is likely to be infrequent, the HIV reservoir represents only a small fraction of all infected cells, and the contribution of direct infection to long-lived reservoirs is under active investigation.

### 1.2. Response to Antiretroviral Therapy In Vivo

**Figure 2:**
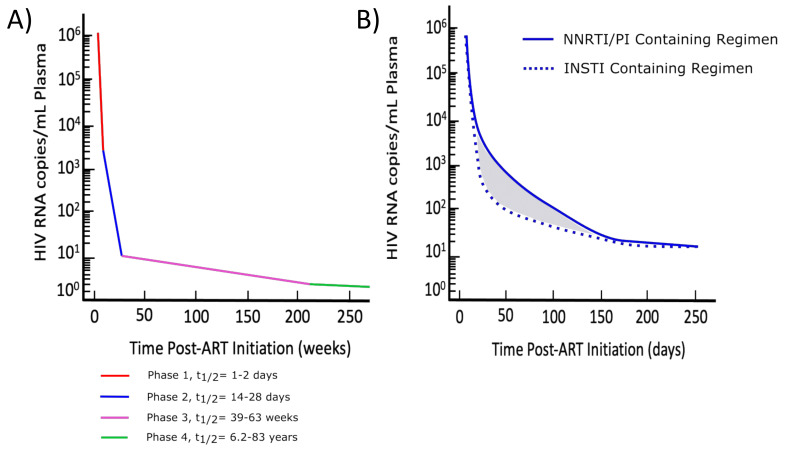
(**A**) Multiphasic model of viral decay. The four phases of HIV RNA decay after starting ART are shown. (**B**) Phase 1 viral load decay is greater in INSTI-containing regimens. The greater rate of phase 1 decay is likely due to the existence of two distinct populations of HIV-infected cells, some that integrate DNA slowly, and others that integrate more quickly. HIV RNA rapidly declines as INSTIs prevent integration in faster-integrating cell populations. This trend may also be explained by the later stage in the HIV lifecycle at which integrase inhibitors act. (Adapted from [65].).

An analysis of the response to antiretroviral therapy (below) has yielded critical information regarding the cells producing HIV RNA and those comprising the latent reservoir. ART controls HIV production by targeting the specific steps of the lifecycle. ART is frequently initiated during chronic HIV infection when viral levels are at a stable steady state. In response to ART, peripheral viral RNA levels undergo predictable sequential decreases dependent on the half-life of the infected cells (see Figure 2) [65]. In the 1–2 weeks after the introduction of ART, HIV RNA declines by 10- to 100-fold, corresponding to a >90% decrease in the viral load. The half-life (c. 1–1.5 days) reflects the half-life of the activated cells responsible for virus production. These data demonstrate that the great majority of all of the virus produced is derived from cells with a short half-life, likely highly activated T cells. On the basis of modeling, the decay curve is next driven by a population of cells with a half-life of approximately 28 days. These longer-lived HIV-infected cells may be macrophages, or partially activated lymphocytes, which have a similar half-life. This second phase of decay is slower than phase one and lasts several weeks. At this point, clinical suppression (viral load less than the limit of assay detection, typically less than 20 copies/mL plasma) is frequently achieved, commonly within six months of initiating ART. The development of sensitive single-copy assays enables the detection of viremia even after prolonged suppressive ART. The internally controlled real-time reverse-transcriptase-initiated polymerase chain reaction (PCR) assay that quantifies HIV-1 RNA with single-copy sensitivity can detect HIV at low levels, generally limited only by the sample volume; levels of 1 copy/10 mL plasma are achievable, or those that are 200-fold more sensitive than the commercial limits [66,67,68,69]. Through the use of single-copy methods, a third phase of HIV RNA decay, with an estimated half-life of 39–63 weeks, was reported. More recently, a fourth phase of decay was detected, with a half-life between 6.2 and 83 years, based on findings from a longitudinal cohort study [70]. Latently infected CD4 T cells, including expanded clones, likely contribute to phases 3 and 4, on the basis of their prolonged half-life. [65,70,71,72,73]. The multiphasic model of viral decay is shown in Figure 2A.

The kinetics of viral load decay can be affected by the presence of integrase strand transfer inhibitors (INSTIs) in a combination ART regimen. At the time of INSTI initiation, some cells will have completed reverse transcription, but not yet integration, while others will already have an integrated provirus. On the basis of this model, INSTI-containing regimens have demonstrated faster decay kinetics than non-INSTI-containing regimens after the initial first-phase decay, as shown in Figure 2B. The more rapid viral load decay with INSTIs is attributable to the ability of INSTIs to block HIV infection, even after reverse transcription has taken place. In ART regimens without INSTIs, cells that have completed reverse transcription will undergo integration and subsequent virus production. In contrast, in the presence of INSTIs, those cells that completed reverse transcription can still be blocked from integration and subsequent virus production [74,75,76]. This faster decay rate with INSTIs should not be interpreted as higher drug efficacy. There is currently no evidence indicating that variation in decay kinetics associated with the initiation of different ART regimens impacts clinical outcomes.

HIV can develop resistance to INSTIs and other antiretrovirals (ARVs) when drug levels are not adequate to suppress the virus, most commonly because of missing doses [77]. Resistance to antiretroviral drugs can be transmitted or acquired. Transmitted drug resistance occurs when an individual is infected with virus that is already resistant to a particular drug or drugs. Acquired resistance occurs when a resistant strain emerges after a patient has been on ART. Resistance-conferring mutations can occur by the substitution, insertion, or deletion of nucleic acids that code for amino acids that comprise the proteins of HIV [78,79]. ARVs within the same class often share resistance-associated mutations; for instance, if a patient harbors a virus resistant to one non-nucleoside reverse transcriptase inhibitor (NNRTI), it is possible that they will be resistant to other NNRTIs. Resistance mutations are archived and must be taken into consideration when designing ART regimens, even when the mutations are no longer detected by standard genotyping [80,81]. Furthermore, resistant mutants may enter the long-lived HIV reservoir, creating an additional potential challenge to cure approaches.

INSTI resistance is of particular interest, as INSTIs are part of all current generally recommended first-line HIV-1 treatment regimens [82]. Mutations can occur at the INSTI binding site, at other locations to counteract the fitness cost of primary mutations [83], and at polymorphic positions related to the subtype and geographical distribution [84]. Three major pathways to raltegravir resistance have become evident, namely, the mutations Q148H/K/R, Y143C, and N155H [84,85]. Table 1 shows the currently available INSTIs and their half-lives and select resistance mutations.

## 2. Potential Mechanisms of HIV Persistence

**Figure 3:**
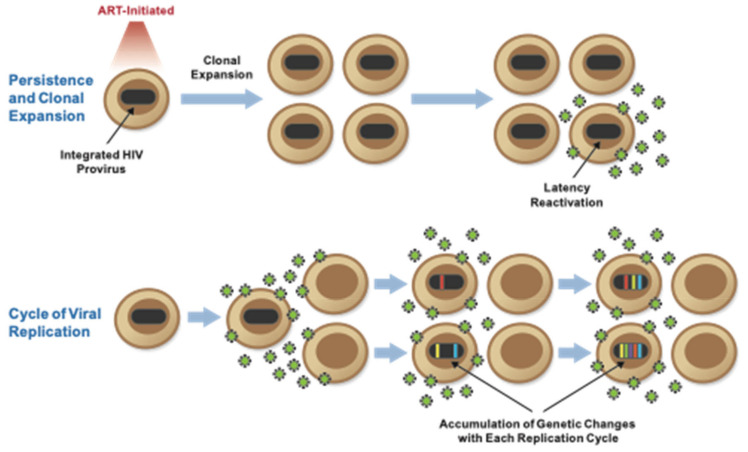
Clonal expansion vs. viral replication. HIV infection may persist via multiple mechanisms. Without ART, or with inadequate ART, cycles of active viral replication continue, leading to the infection of new susceptible cells. New cycles of infection result in new mutations and genetic variation. Even while on effective ART, HIV proviruses persist via the clonal expansion of HIV-infected cells. Clonally expanded cells may be replication-competent, periodically reactivate from a latent state, and may produce infectious virus, leading to low-level viremia. With effective ART, this low-level viral production is unlikely to infect new cells. Adapted from [87].

As described above, the recognition that ART did not result in a sterilizing cure for HIV engendered the search for a source of the virus able to rebound after being undetected by the available assays. A number of potential mechanisms have been reported to explain HIV persistence, chiefly divided into models that require low-level ongoing cycles of HIV replication, as well as those that require long-lived persistence and the clonal expansion of cells that were infected prior to the introduction of ART (Figure 3).

### 2.1. Ongoing Cycles of HIV Replication

In this model (Figure 3), ART blocks most, but not all cycles of HIV replication. The inadequate penetration of antiretrovirals permits replication and new cycles of infection in sanctuary sites where HIV-infected cells are present, such as the central nervous system, the gut, or lymphoid tissue. The consequences of ongoing replication include the presence of 2-LTR circles in infected cells, as well as the ongoing accumulation of genetic change in viral genomes with molecular evolution, despite long periods of ART. As such, the HIV reservoir undergoes genetic change during ART, and the genetic composition of the rebound viremia after interrupting ART will have evidence of molecular evolution.

### 2.2. Persistence and Clonal Expansion of HIV-Infected Cells

In this model of HIV persistence, ART effectively blocks ongoing replication. As a result, no new cycles of HIV replication take place, and no molecular evolution of HIV is present during long-term ART. Instead, infected cells persist and can undergo clonal expansion. As such, the HIV reservoir is maintained by cell division, not virus replication. Low-level HIV viremia results from the production of HIV from these long-lived cells and their daughters. The consequences of persistence and clonal expansion are that rebound viremia will have the genetic characteristics of HIV present prior to initiating ART, without evidence of molecular evolution. LTR forms of HIV may persist because of their long half-life as episomes, but their numbers will be diluted as the infected cells undergo division.

### 2.3. Implications of Persistence Mechanisms

The above mechanisms of HIV persistence are fundamentally different and require distinct cure strategies to eradicate HIV. If ongoing replication is the source of persistent viremia, improved ART could be expected to have an impact on the HIV reservoir and HIV eradication. In contrast, if long-lived cells capable of clonal expansion are responsible for HIV persistence, new strategies of HIV eradication will be necessary. As such, it is critical to determine the primary mechanism of HIV persistence, and several clinical and laboratory approaches have been developed to address this fundamental issue.

## 3. Laboratory Approaches to Studying Persistence

### 3.1. Nucleic Acid Analyses and Bioassayss

During the course of the study of HIV persistence, a number of laboratory approaches have been developed to characterize and quantify HIV-infected cells. Others have described and reviewed many of these techniques [18,19,21,88,89,90]. In general, these assays can be categorized as assays quantifying HIV nucleic acid (DNA or RNA) or measuring levels of biologically active HIV by detecting HIV using an assay measuring the replication-competent virus. These techniques rely on clinically derived specimens.

In terms of quantitative nucleic acid measurements, the most common assays are PCR assays targeting the conserved regions of the HIV genome. These include quantitative PCR (qPCR), which is a probe-based assay utilizing fluorescent dyes to target sequences of interest, and digital droplet PCR (ddPCR), which partitions PCR reactions into thousands of individual oil droplets, consequently resulting in the absolute quantification of HIV DNA [91,92]. In individuals with suppressed plasma viremia below the limit of detection of commercial assays, highly sensitive single-copy assays can be used to detect low levels of HIV RNA in plasma [66,67,68,69,93]. There are also single-copy assays that can detect HIV DNA and RNA within cells, referred to as cell-associated DNA and RNA [94,95]. Other single-cell approaches include branched DNA signal amplification, sequencing (e.g., DNAseq and RNAseq), and virus barcoding [96]. Single-cell RNA fluorescence in situ hybridization-flow cytometry (FISH-flow) assays can be used to detect subpopulations of CD4 T cells that transcribe HIV RNA within each T-cell subset [97]. Another single-cell technique is cell-associated RNA and DNA single-genome sequencing (CARD-SGS), which can determine the levels of RNA expression from proviruses in single cells and quantify HIV RNA in infected cell clones [98]. The novel HIV-1 SortSeq method identifies one HIV-infected cell per million uninfected CD4 T cells by inducing latency reversal with phorbol 12-myristate 13-acetate (PMA) and ionomycin ex vivo. The results from HIV-1SortSeq have been correlated with the latent reservoir size by a quantitative viral outgrowth assay (QVOA) [99]. Tissues and tissue microenvironments can now also be analyzed for HIV and DNA quantification, using modern in situ hybridization techniques [100]. Multiplexed immunofluorescence and in situ hybridization (mIFISH) allow for the quantification of HIV DNA and RNA in situ from human tissues, with the identification of the cellular source of fluorescent signals [101].

Nucleic acid measurements have distinct advantages and disadvantages. These assays readily allow for the quantification of HIV DNA and RNA, are applicable to both tissue and plasma samples, are highly sensitive at the single-copy level, and can be combined with an analysis of the sequences of HIV nucleic acids. Additionally, these measures can be studied as correlates of other soluble and lymphocyte markers of immune activation. On the other hand, these techniques are not able to differentiate between replication-competent and -incompetent proviruses, nor do they provide information on how measured nucleic acids contribute to the reservoir.

Other assays focus specifically on replication-competent proviruses. The QVOA provides an important minimal estimate of the replication-competent reservoir size through the culturing of purified CD4 T cells from a donor of interest with phytohemagglutinin-activated peripheral blood mononuclear cells from HIV-uninfected donors. This exponentially expands the HIV virus and allows for an estimation of the frequency of replication-competent virus [21]. QVOA is known to underestimate the replication-competent reservoir, given that it will miss proviruses not induced by a single round of T-cell activation, and is dependent on the conditions used to stimulate viral outgrowth [102]. A number of modifications of the QVOA assay have been developed to increase the sensitivity (see [21] for review).

The intact proviral DNA assay (IPDA) also quantifies replication-competent proviruses through ddPCR, which separately quantifies genetically intact proviruses [103,104]. The two amplicons utilized are strategically placed in the conserved regions of the proviral genome (packaging signal and Rev-response element of the *env* gene), which are used to infer whether a provirus is defective or not. An intact (replication-competent) provirus is positive for both signals. It produces larger estimates of the intact proviral reservoir than QVOA and is considered by some to be more accurate because, unlike QVOA, it does not depend on the expression status of the proviruses. This method has been used to compare the decay of intact and defective proviruses on ART [104].

### 3.2. Genetic Characterization of HIV Variants

In addition to proviral quantification, techniques have been developed to characterize proviral populations because the genetic diversity of HIV sequences is relatively high in PLWH because of rapid and error-prone HIV replication. This is due to reverse transcription being highly error-prone [105]. If ongoing replication were occurring, high rates of recombination and new viruses should be seen over time. Genetic analyses can be used to determine how viruses are related, including whether they are clones, and they have been employed to explore this possibility. Phylogenetic evaluation will reveal identical sequences and integration sites in different cells if clones are present. An assessment of the diversity in different anatomic locations also reveals whether compartmentalization is occurring, which would result in different sequences accumulating at different anatomic sites. The neighbor-joining and maximum likelihood approaches, used to estimate the phylogenetic distance between viral RNA or proviral DNA variants, are the most common methods used. Neighbor-joining trees facilitate the quick visualization of sequence diversity and require few assumptions to create. By contrast, maximum likelihood trees allow more robust inferences about evolutionary relationships and compartmentalization, while requiring more underlying assumptions about the molecular evolution taking place [106,107]. In general, these methods correlate well [107].

The phylogenetic characterization of HIV genetic diversity and evolution on suppressive ART has demonstrated that it is the proliferation of infected cells, rather than ongoing viral replication, that maintains the reservoir. However, early studies were interpreted as supporting a significant role for the cycles of replication [108]. A subsequent study of DNA-level phylogenetics in tissues obtained, at colonoscopy or autopsy, of patients on ART for at least eight years found the clonal expansion of infected cells present prior to the start of ART, but no evidence of ongoing replication or compartmentalization [3]. Several other studies support this conclusion and are discussed below. 

Phylogenetic analyses in chronic untreated patients have also revealed an exchange of the virus and infected cells between the blood and lymphoid compartments. This was inferred on the basis of the genetic similarity of proviral DNA in memory and naïve CD4 T cells from lymph nodes and blood, as well as HIV RNA from plasma [109,110]. The sequencing of individual DNA molecules from single CD4 T cells in a lymph node and peripheral blood showed that >90% of infected cells contain only one copy of HIV DNA, which implies a low potential for productive recombination [110,111]. Additional analyses have indicated that a small number of cells (c. 1%) that have two proviruses could be responsible for the observed level of recombination [41,110,111].

While phylogenetic analyses are extremely informative about HIV reservoirs, they do have limitations. They are only as good as the available data, which is incomplete because patient specimens only sample a fraction of the viral variants present in the patient, and generally only a portion of the genome. Phylogenetic trees are also inferred on the basis of specific assumptions about sequence relationships and do not reflect the viral phenotype. While certain characteristics can be extrapolated from genetic sequences, the viral function is not actually being assessed.

### 3.3. Analysis of Integration Sites

The characterization of clonal expansion has been instrumental to our understanding of HIV persistence. A number of useful assays have been developed to identify and quantify HIV integration sites, which have been reviewed in detail [52,64,112,113]. These early assays identify precise integration sites for HIV, but do not provide an extended sequence of the HIV provirus. Several new assays, reported to be capable of extended proviral characterization, have now been described. Integration site analysis allows for the differentiation between clonal expansion and the PCR artifact. Identical integration sites found to have different lengths of host sequence are likely the product of clonal expansion, while identical integration sites, with identical host sequence lengths, are likely the products of PCR amplification [52,64,114]. With the growing interest in characterizing integration sites, open-source public repositories for integration sites (Retrovirus Integration Database (RID) (https://rid.ncifcrf.gov accessed on 29 November 2021) and for proviruses (Provirus Sequence Database, https://psd.cancer.gov/ accessed on 29 November 2021) have been developed. New approaches modeling the emergence and fate of clones are currently under study [115].

All of the approaches used to directly assess integration sites and clonal populations have limitations. For example, integration site identification does not include the sequence of the entire provirus, and matching plasma viruses or proviruses of interest to their precise integration sites remains labor-intensive. Proviral sampling is limited by the ability to amplify all of the LTR sequences. HIV integration sites have typically been evaluated by PCR, employing a primer complementary to LTR that is based on the consensus sequence of the LTR of HIV from each study participant. Targeting LTR with a single primer for amplification may not sample proviruses with genetic variations in LTR, especially in the U3 domain of LTR, and new approaches to improve sampling are underway, including the use of a pool of capture probes to enrich the sampling of genetically diverse LTRs [115]. The efficiency of integration site analysis remains suboptimal, though new approaches are being developed [116,117,118]. Current analyses are estimated to capture c. 10% of the proviruses present in a sample. Furthermore, a sample size (10–20 million cells) represents only a small proportion of the infected cell population. As an example, an analysis of 1000–2000 integration sites suggests that c. 40% of all proviruses were present in clones. A study of much larger databases suggests that the fraction of cells in clones may be much higher [51]. It has been hypothesized that the majority of, if not all, HIV-infected cells in individuals on suppressive ART are members of clones [119]. Consequently, very large sample sizes are essential to accurately quantify the sizes of clones, and the changes in individual clone sizes, during long-term ART.

## 4. Clinical Approaches to Study Persistence

### 4.1. Natural History Studies

In addition to laboratory assays, clinical approaches have been useful in investigating the HIV reservoir. Both natural history and interventional protocols have been utilized, beginning with early cross-sectional and longitudinal investigations of T cells and antiretrovirals. Among the potential cellular and anatomical reservoirs contributing to persistence, the greatest contributor is thought to be latently infected resting memory CD4 T cells carrying proviral DNA. In 1995, Chun et al. demonstrated, through the isolation of resting CD4 T cells and the selective amplification of integrated HIV-1 DNA, that an integrated viral reservoir also exists within resting CD4 T cells, thus confirming a far more long-lived and durable mechanism of viral persistence [59,60,61]. Subsequently, a study of patients on effective ART found that the frequency of resting CD4 T cells harboring replication-competent latent HIV-1 was low (0.2 to 16.4 per 10^6^ cells). Nonetheless, replication-competent integrated provirus, particularly in resting and activated CD4 T cells and macrophages, was thought to be able to sustain HIV infection [62]. Cross-sectional analysis revealed that these cells did not decrease during long-term ART. Furthermore, the recovered viruses generally did not have mutations associated with resistance to the patient’s ART [62,120].

Soon after the discovery of latently infected T cells, Finzi et al. demonstrated that this could provide a mechanism for the lifelong persistence of HIV-1 [61]. As the half-life of this reservoir was estimated at 44 months, implying that the eradication of this slow-decaying reservoir would require 73.4 years on ART, ART alone could no longer be considered a viable strategy for the cure of HIV infection. Chun et al. examined resting CD4 T cells in both the blood and lymph nodes to quantify latent tissue reservoirs in comparison to peripheral blood [59]. They estimated that replication-competent proviruses existed in only 5–7 resting CD4 T cells per 10^6^ cells in lymph nodes and blood. Their estimates of latently infected CD4 T cells exceeded the estimates of infection with replication-competent proviruses, providing early evidence of the largely defective proviral populations that accumulate during long-term ART.

More recent natural history studies continue to elucidate the dynamics of the HIV reservoir. Such studies include the Swiss HIV Cohort Study, the Zurich Primary HIV Infection Study, and SEARCH 010, which demonstrated that early ART initiation in acute infections decreases the plasma of the HIV-1 RNA setpoint on ART [121,122,123,124,125]. Therapy during acute infection is associated with improved immune recovery [126]. Subsequent studies have suggested that reservoir size may, in part, be determined by genetic factors, while clinical observations, such as viral blips and low-level viremia, are associated with slower reservoir decay [127]. Studies demonstrating that the loss of replication-competent HIV and increases in the proportions of *gag*-deleted provirus increase during the disease course suggest that immune responses shape proviral populations [104,128,129].

### 4.2. Interventional Studies to Assess Ongoing Replication

While ongoing virus production and reservoir persistence were clearly demonstrated in the setting of peripheral suppression below the limits of clinical assay detection, questions about the role of ongoing replication cycles in maintaining low-level viremia remained. Interventional studies have been utilized to investigate the potential for ongoing replication through several methods, including ART intensification studies, latency reactivation studies, and ART interruption studies. If ongoing replication occurs during ART, it should, theoretically, be possible to further suppress viremia with more effective ART, or an ART that can more readily reach potential anatomic sanctuaries. The effectiveness of ART might be improved by the intensification of a current regimen, either by increasing the dose of the component drugs or by the addition of another effective drug. A number of intensification studies have been performed (See Table 2 and [130]). Intensification has not produced complete suppression in patients with low-level viremia on effective ART. In 2010, Buzón et al. demonstrated that the addition of the integrase inhibitor, raltegravir, to a combination ART regimen resulted in a transient increase in 2-LTR circles, suggesting that virion generation and de novo infection were potentially occurring, despite a suppressive ART regimen [131]. Because they impair the integration of viral DNA into host DNA, INSTIs can result in the accumulation of unintegrated circular viral DNA forms, including 2-LTR circles [132,133]. Among ART intensification studies, the most definitive study to demonstrate that the ongoing replication and the infection of new cells is an unlikely contributor was a randomized double-blind placebo-controlled trial of 40 patients on suppressive ART that underwent the addition of a placebo or dolutegravir to their regimen. The primary outcome was a change in the frequency of the 2-LTR circles in the peripheral blood of CD4 T cells. The median 2-LTR circle fold-changes, from baseline to day 7, were −0.17 (IQR −0.90 to 0.90) in the dolutegravir group, and −0.26 (−1.00 to 1.17) in the placebo group (*p* = 0.17). Since the accumulation of 2-LTR circles should occur if the ongoing replication was blocked by the INSTI, the authors concluded that ongoing replication is rare [11]. Numerous additional interventional studies have been conducted to address this possibility, generally supporting the same conclusion and providing insights into the factors affecting the markers of immune function. They are summarized in Table 2.

Latency reactivation studies seek to induce viral replication in latently infected cells through latency-reversing agents, thereby leading to the death of those cells. In theory, the complete reactivation of HIV-infected cells could eradicate the HIV reservoir, as long as all the activated cells undergo cell death without undergoing clonal expansion. While latency reversal has been demonstrated as possible, such interventions have failed to reduce the reservoir size [88,156,157,158,159]. Latency reversal is discussed in further detail under the “Implications of Persistence for Cure Strategies” section of this review.

Analytic treatment interruption (ATI) studies have also been used to assess the HIV reservoir. In such studies, individuals who have previously been on ART discontinue their ART regimens while their viral loads and reservoir dynamics are monitored. ATI studies have led to the discovery of several important characteristics about the HIV reservoir and the potential for ongoing replication. Most individuals experience viral rebound, demonstrating the re-emergence of replication-competent virus [160,161]. However, a small number of individuals do not experience viral rebound after ATI and they are referred to as "post-treatment controllers" [162]. No unique source of viral rebound has been found. Rather, the sources of the rebound virus may be any cellular or tissue compartment harboring replication-competent proviruses, thus emphasizing the importance of anatomic reservoirs.

Joos et al. found that rebounding virus, after 2 weeks of ATI, was homogenous, suggesting the stochastic reactivation of latently infected clones as opposed to viral populations undergoing low-level replication. Population diversity increased steadily and achieved pretreatment levels >2.5 years after ATI, on average [163]. Rothenberger et al. compared the number of rebounding/founder variants in the lymph nodes, gut-associated lymphoid tissue (GALT), and plasma of patients undergoing ATI. Sequencing demonstrated many rebounding/founder viruses representing recrudescent viremia from multiple sources, implying the reactivation of many latently infected cells from multiple sites. Initially, the rebound virus was genetically diverse, and this was followed by the establishment of a dominant virus [164]. Bednar et al. found a high diversity of viral env and tropism for CD4 T cells, as opposed to myeloid cells, in the peripheral blood samples of patients undergoing ATI [165]. A comparison of latent viruses from CD4 T cells in peripheral blood and lymph nodes to those emerging during ATI showed a 98% overlap of intact or replication-competent clonal sequences between the blood and lymph nodes; however, there was no overlap between 205 latent-reservoir and 125 rebound sequences in four individuals who underwent ATI. Recombination was implicated, supporting its role in rebound viremia [166]. Virus in the blood may be different from that in the tissues during rebound given the differences in the contributing cell types, the degree of compartmentalization, and the opportunities for recombination. It is likely that clonal expansion plays a major role in viral rebound and serves as a major target in preventing it [167]. Some rebound variants have also been identified as genetically identical to viruses detected during ART, which suggests that viral evolution and ongoing replication were not taking place during ART [168]. While the predictors of the time to viral rebound continue to be elucidated, it is likely that they are associated with baseline or interval viral loads [169,170,171]. Some potent and broadly neutralizing antibody therapies, such as VRC01, can slightly delay, but not prevent, viral rebound during ATI [172]. A historical look at AIDS Clinical Trials Group (ACTG) data shows that early ART initiation, regardless of regimen, was associated with a delay in the time-to-rebound viremia after the initiation of ATI [173]. Importantly, ATI does not appear to lead to an increase in the size of HIV proviral populations [174].

A number of laboratory and clinical studies have addressed the source of HIV persistence during ART. The lack of an intensification effect on the levels of HIV viremia, together with little or no evidence of molecular evolution in persistent low-level viremia during ART, or in viremia that re-emerges when ART is interrupted, indicate that the persistence of HIV is not due to ongoing replication. Notably, this conclusion relies on the absence of evidence. These studies cannot rule out that rare transmission events can occur during ART. There is no evidence, however, that such unlikely events sustain the reservoir in the presence of large populations of long-lived and clonally expanded cells. As such, eradication strategies have been focused on the long-lived population of HIV-infected cells.

## 5. Proviral Landscape during ART

### The Proviral Landscape Undergoes Dynamic Change during ART

The finding that HIV does not accumulate new genetic changes during ART does not imply that the HIV proviral population is static. In fact, accumulating evidence demonstrates that the HIV proviral population undergoes ongoing change in the context of host–virus interactions [175], even from the onset of infection. The stage of infection when ART is initiated may affect the number of HIV-infected cells that are present during long-term ART; initiating ART within weeks of infection may result in lower levels of cell-associated HIV DNA [176,177]. In addition, elite controllers generally have lower levels of HIV proviruses [178]. Once ART has begun, the numbers of infected cells decline 10- to 30-fold until they reach a plateau at approximately 4 years of treatment [179]. While the size of the HIV-infected population reaches a relatively stable level, its composition continues to undergo dynamic changes during ART. In general, intact proviruses are lost over time while the relative proportion of deleted proviruses increases. This has been demonstrated using assays that detect potentially replication-complement proviruses (IPDA) and that have shown that intact proviruses are lost faster than defective proviruses [104,129]. This represents a shift in the population as opposed to overall expansion. While it is not clear if 5′- or 3′-deleted proviruses are preferentially selected, mutations, insertions, or deletions could contribute to proviral defects.

In addition, the relative levels of proviruses with mutations or deletions increases during ART [128,129]. The relative enrichment of deleted proviruses did not occur during the first- or second-phase decay of viremia, but only after 2–3 years of ART [128]; it is possible that intact replication-competent, or *gag*-containing, proviruses are actively eliminated when they express HIV proteins that are immunogenic. While *gag* is the best studied example, other HIV proteins may also be immunogenic. HIV *gag* is a commonly studied example because there are epitopes in *gag* that are highly conserved and that elicit strong CTL responses, some of which have been associated with the natural control of HIV [180,181,182,183]. The active elimination of expressed proviruses may explain the recent finding that proviruses in elite controllers are located in poorly transcribed centromeric regions [15]. It is likely that HIV integrates widely in cells, as in those of noncontrollers, but the effective immune elimination of many cells that are expressing HIV results in a relative enrichment in the proportion of proviruses in areas with little transcription, such as the centromeric regions of the genomes.

Additional forces driving changes in the proviral landscape are poorly understood. Studies have typically focused on peripheral blood samples, which may not be an accurate reflection of the dynamic changes in the total HIV reservoir. Nonetheless, the characterization of the populations in the peripheral blood offers insight.

**Figure 4:**
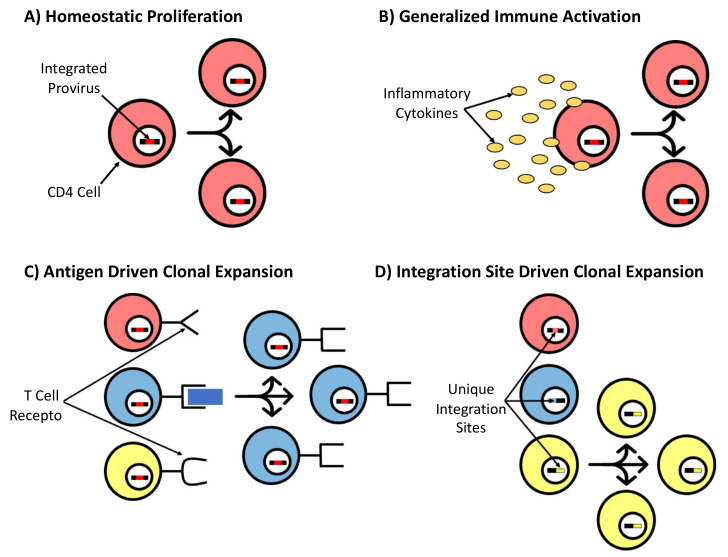
Mechanisms of clonal expansion. (**A**) Homeostatic proliferation maintains existing CD4 T cells. (**B**) Generalized immune activation can stimulate CD4 T-cell proliferation. (**C**) Antigen-binding to a CD4 T-cell receptor can stimulate clonal expansion. This stimulus for clonal expansion can create larger clones than the other mechanisms. (**D**) In rare instances, the site of proviral integration into the host genome can stimulate clonal expansion.

As ongoing cycles of replication are blocked by ART, no new cells are entering the population of infected cells, and changes in the proviral landscape are the result of changes in the populations of infected cells present at the time ART is introduced. Infected cells essentially have three broad fates: they may be eliminated, persist as single cells, or undergo clonal expansion. Persistence is clearly favored by clonal expansion. The clonal expansion of HIV-infected cells can be detected within weeks of HIV infection and likely takes place throughout infection [184]. It has been estimated that over 40% of all cells with proviruses are clonally expanded [52,112], and this proportion may be much higher [185]. The proliferation of the resultant clonal populations may be homeostatic because of generalized immune activation, either antigen-driven or integration-site-dependent. These mechanisms are influenced by viral, immunologic, and genomic factors [175] and are conceptually illustrated in Figure 4. These mechanisms vary in their contributions to the development of clonal populations, with some mechanisms contributing heavily and others only in limited circumstances [184]. All mechanisms permit the expression of viral proteins, but do not necessarily predispose to such expression. When proteins are expressed, additional clonal expansion may be inhibited by an immune response.

The forces driving antigen-specific CD4 T-cell expansion are not well-defined but are likely to include antigen exposure, cytokine profiles, and generalized immune activation. The proliferation of latently infected cells also includes homeostatic proliferation [186]. This occurs without the production of the virus and contributes to reservoir stability. Furthermore, the sequencing of replication-competent proviruses at different time points revealed that some clones wax and wane [187]. Recurrent antigen exposure has been shown to influence clonal selection and expansion in patients who are both HIV- and Cytomegalovirus (CMV)-positive. Simonetti et al. used CMV lysates, *gag* peptides, and control αCD3/28 antibodies to stimulate the proviruses of PBMCs from patients co-infected with HIV and CMV. They found a higher proportion of identical sequences and higher clonality by integration site analysis in CMV-specific cells versus controls. Identical sequences were less abundant in *gag*-specific cells and most clones harbored defective provirus. Viral outgrowth from CMV-specific cells of one subject matched the results from 8 months prior, suggesting the persistence of a clone harboring infectious provirus selected in response to CMV [188]. Another study assessed the responses of reservoir cells to antigens from the common viral causes of chronic or recurrent infections, including *gag*, CMV, EBV, influenza, and tetanus toxin, in ART-suppressed individuals. Clones of antigen-responsive CD4 T cells containing defective or intact viral DNA were identified in seven out of eight subjects. Central, transitional, and effector memory T cells, which have undergone antigen-stimulated division, contain expanded clones of HIV integrants [189]. The same group also showed that expanded clones tend to contain defective provirus, consistent with other studies, and that clones expand with time on suppressive ART [112]. The production of HIV proteins by these expanded clones may engender immune activation. The authors note that these findings are consistent with reports of viral blips being more common during winter, when seasonal viral infections are more frequent [189]. These studies suggest that chronic or repeated antigen exposure stimulates clonal expansion, contributing to the maintenance of the reservoir.

Even when clones do not harbor infectious virus, they can produce viral RNA and protein that may serve as sources of activation [7,9,190,191]. Immune activation persists despite ART, which has been demonstrated by soluble inflammatory markers, such as interleukin (IL)-6 and soluble CD14 [192,193]. This is of clinical importance because chronic inflammation and immune activation have been linked to morbidity and mortality during long-term ART [194]. Efforts to reduce HIV-associated chronic immune activation with immunomodulators, such as with ruxolitinib, have shown potential [195]. The association between immune activation, clonal expansion, and the increased prevalence of defective proviruses as ART duration increases remains uncertain but is under investigation [196].

In response to the depletion or expansion of T-cell populations, the immune system largely restores homeostasis via cytokine signaling. IL-7 and IL-15, and, to a lesser extent, IL-2, IL-10, IL-12, interferons, TGF-ß, and transcription factors have been implicated in the survival of CD4 and CD8 T cells [197,198,199,200,201]. Chomont et al. identified central memory and transitional memory T cells (T_CM_ and T_TM_) as major HIV cellular reservoirs in ART-treated patients and showed that proviral DNA is detected in T_TM_ from aviremic patients with low CD4 T cells and high IL-7-mediated homeostatic proliferation [186]. By modeling antigenic stimulation and homeostatic proliferation in vitro, Bosque et al. showed that IL-2 plus IL-7 induces the partial reactivation of latent HIV but that it cannot reduce the reservoir size. Furthermore, latently infected cells can homeostatically proliferate without reactivation or cell differentiation. Conversely, antigenic stimulation reactivated provirus in T_CM_ and induced the depletion of latently infected cells by virus-induced apoptosis. Thus, IL-7 may stimulate the proliferation of memory cells, which counteracts its ability to purge latent virus [200]. The lack of reactivation during homeostatic proliferation presents a challenge to eradication, as this does not induce viral expression or immune clearance. It is important to note that cytokine stimulation plays a role in both homeostatic proliferation and generalized immune activation, for both involve stimulation by other immune cells.

Generalized immune activation, arising from insults to the mucosal integrity, may drive, or derive, from clonal expansion. For example, HIV, or other infections, may damage the integrity of the gastrointestinal mucosa, allowing for the translocation of microbes and their products. Along with the HIV, microbial products may nonspecifically (e.g., via LPS) stimulate the activation of T cells and the expansion of CD4 T cell populations, including clones. Expanded populations may produce more virus or viral products that perpetuate the cycle of mucosal damage and the resultant generalized activation [201,202].

A final mechanism driving clonal expansion is the presence of the provirus itself. As described above, HIV integrates largely in regions of active transcription, and typically in an orientation-independent fashion. In an exhaustive analysis of >50,000 proviral integration sites, Coffin and coworkers demonstrated that infected cells may undergo selection because of the presence of the provirus [51]. Although HIV has a preference for genes undergoing active transcription, cells with proviruses in very highly expressed genes are negatively selected [51]. It is possible that the increased transcription of HIV in this setting may target infected cells for elimination. In addition, although integration takes place in an orientation-independent fashion at the time of infection, with time on ART, there was an increase in the relative abundance of cells with proviruses oriented in the opposite orientation of the host gene, indicating that cells with proviruses in the same orientation are under negative selection [51]. In rare cases, proviral integration in specific genes drives persistence. For a series of seven genes, (*BACH2, MKL2, MKL1, STAT5B, MYB, IL2RB,* and *POU2F1*), HIV integrates widely and may be present in either orientation. After prolonged therapy, however, only a subset of integrants remain, concentrated in restricted areas of the respective genes, and always in the same orientation as the host gene. These data indicate that the presence of proviruses in a subset of introns, and in the same orientation as the host gene transcription, drives the persistence and expansion of infected cells [51,52,112,113,203]. An analysis of these rare, but dramatic examples of persistence and clonal expansion will shed new light on the mechanisms of persistence.

These mechanisms of clonal expansion contribute to the maintenance of the HIV reservoir without invoking new cycles of infection. They can all occur in patients on effective ART and may evade immune detection. Clonal populations not only sustain the reservoir but may also contribute to HIV-associated morbidity and mortality through the production of viral antigens that can have direct toxicity or stimulate inflammatory responses [7,188,194,204].

## 6. Persistence in the Context of Infected Individuals

### 6.1. Target Cells and Anatomic Sites

To date, the descriptions of HIV populations are predominantly based on studies of peripheral blood cells, yet the majority of HIV-infected cells are located in tissues. An understanding of HIV-infected cell populations in these different sites is critical for designing cure strategies that seek to eliminate the reservoir. The key features of cell types and the anatomic compartments that harbor the virus are described in this section. HIV virions differentially infect susceptible cell types by targeting specific cells on the basis of expressed proteins. Thus, a virus housed in different anatomic compartments and cell types may vary in quantity, lineage, replication competence, insertion site, and activity level. In the context of persistence, rebound virus during ART cessation may differ depending on what anatomic compartment harbors the repopulating virus. Cure strategies may need to address viral variants harbored in distinct body compartments.

**Figure 5:**
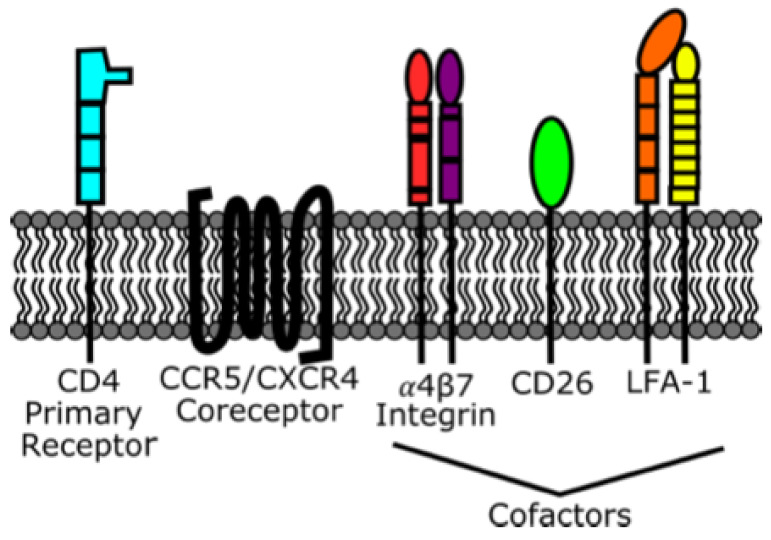
Receptors and coreceptors on CD4 T cells. Tropism of HIV is determined by receptors and coreceptors that allow for binding to the cell surface. LFA-1: lymphocyte-function-associated antigen. Adapted from [207].

CD4 T cells are the most commonly infected cell type because viral gp120 specifically binds the CD4 receptor. Once the CD4 binding site of gp120 has attached to CD4, gp120 will also bind either the CC chemokine receptor type 5 (CCR5) or CXC motif chemokine receptor 4 (CXCR4) coreceptor, as shown in Figure 5. Viruses are classified as "CCR5 tropic", "CXCR4 tropic", or "dual/mixed tropic" on the basis of the coreceptor they use. CCR5 tropic viruses are known as “R5 viruses" and CXCR4 tropic viruses are known as “X4 viruses”. X4 and dual-tropic viruses have also been associated with the later stages of infection. Tropism plays a role in treatment decisions, for CCR5 antagonism is an ARV (maraviroc) mechanism of action. Great heterogeneity exists, even within CCR5 tropic viruses, with preference for the infection of particular tissue types, such as gut-homing, skin-homing, and lymph-node-homing [205]. The subset of memory CD4 T cells that reside in lymph node B-cell follicles, termed "T_FH_ cells", carry a higher burden of HIV infection than other CD4 T-cell subsets and are a major HIV reservoir in untreated and ART-treated individuals [206]. In addition to HIV receptors, cofactors on CD4 T cells, such as CD26, α4β7 integrin, and lymphocyte-function-associated antigen (LFA-1) may play roles in facilitating infection [207].

Thus, macrophages can be infected early during infection and may serve as reservoirs. Though macrophages are more resistant to HIV infection than CD4 T cells and generate fewer virions, they can establish infection at mucosal sites and carry the virus to other locations, such as the central nervous system (CNS), lymphatic tissues, and the gastrointestinal tract [208]. The decreased susceptibility to antibody neutralization [209] and the ease of crossing the blood–brain barrier facilitate the persistence of infected macrophages in sanctuary compartments.

Antigen-presenting cells, including dendritic cells, are abundant at mucosal sites and can harbor HIV [210,211], although there has been debate about the specific subtype of Langerhans cells [212]. Langerhans cells are tissue-resident macrophages that form a network in the skin epidermis and can migrate to draining lymph nodes. Although they likely serve as immune sentinels, research on their specific role and potential mechanisms of infection is ongoing [213]. Langerhans cells also contain Birbeck granules, which can internalize and degrade HIV through a langerin-mediated mechanism. However, this langerin-dependent barrier can be overcome during inflammation, increasing the susceptibility of Langerhans cells to infection [214,215]. Compared to CD4 T cells, dendritic cells are poorly infected, if at all [216]. HIV may, however, be bound to the surface of dendritic cells and transmitted to T cells by transinfection [217,218]. Transinfection has been demonstrated even in the presence of ART in in vitro experiments, but the presence and role of transinfection in vivo is unknown [218]. In vitro transinfection involves C-type lectin receptors, dendritic cell-specific intracellular adhesion molecule 3-grabbing nonintegrin (DC-SIGN), dendritic cell immunoreceptor (DCIR), and langerin at the surface of dendritic and Langerhans cells, instead of using CD4 and the chemokine coreceptor [207,217,219]. B-cells and granulocytes are also capable of transinfecting T cells using related attachment factors [220,221]. Transinfection may have an important role in establishing reservoirs prior to the introduction of antiretroviral therapy. Binding circulating virions during suppressive therapy may contribute to immune activation and the maintenance of the reservoir [222]. Receptors, coreceptors, and cofactors on CD4 T cells, macrophages, and antigen-presenting cells are summarized in Table 3.

Prior to ART, HIV replication can take place in host cells present in diverse compartments, including the CNS, gut, lymph nodes, and other tissues, such as the skin and lungs. As a consequence, reservoirs can become established in long-lived cells in these sites [226].

### 6.2. Specific Anatomic Compartments with HIV-Infected Cells

Infection and the establishment of viral reservoirs in diverse cell types and anatomic locations present challenges to cure strategies. It is critical to characterize and understand how reservoirs are established and maintained in order to develop approaches for targeting and eradicating the reservoir. Some studies suggest that distinct anatomic sites may be compartmentalized from those found in the peripheral blood or other tissues [227]. While identical sequences were identified in multiple tissues, it remains unclear if these are true clones [118]. Understanding whether identical sequences in different sites are clonal or if they evolved independently may influence cure strategies.

A number of anatomic sites have been described as potential compartments that harbor HIV-infected cells. Here, we will describe infection in lymphoid and nonlymphoid tissues.

HIV infection of the CNS is established by infected cells trafficking across the blood–brain barrier to establish local infection. The virus enters the CNS within days of infection. Long-lived cells, including microglia, macrophages, and astrocytes in the brain can harbor the virus and serve as reservoirs [228]. Prior controversy over whether the CNS actually serves as an HIV reservoir stemmed from doubt about its ability to repopulate other compartments if suppressive ART was stopped. It has now been well-established that HIV can persist in the CNS despite adequate ART in the periphery. The production of the virus in the CNS occurs in the setting of the peripheral suppression on ART [228] and in elite controllers [229]. HIV RNA levels in the CSF can even exceed those of the periphery, which is termed the “CNS escape” [230]. A CNS escape occurs when virions are produced by CNS reservoirs or when they are released from cells that have trafficked into the CNS and have undergone expansion [231].

Replication in the CNS results in genetically distinct HIV variants [232]. These variants derive from two groups: genetically diverse macrophage-tropic populations that represent established CNS infection, and clonally amplified R5 T-cell-tropic populations. The former can enter cells with lower levels of CD4 and decay slowly, while the latter require higher levels of CD4 and decay rapidly. Although microglia and macrophages in the CNS express low levels of CCR5 and CD4, and the infection of these cells in uncommon, infection has been demonstrated in vitro and in vivo [232]. True macrophage-tropic HIV-1 is most often found as a compartmentalized CSF population of HIV capable of infecting cells with low levels of cell-surface CD4 and has been documented in patients with suppressed viremia and individuals with HIV-associated dementia [231,233,234]. These populations are genetically diverse and likely represent established CNS infection. Their presence has been correlated with a slower decay of the virus in the CNS [233]. Nonetheless, a study of paired CSF and blood samples from ART-naïve adults using viruses pseudotyped with Env from 24 subjects showed that all transmitted variants were R5 T-cell-tropic and generally required high levels of CD4 for entry [234].

Virions produced in the CNS are subsequently able to egress to peripheral sites despite suppressive ART [235]. A 2020 study by Chaillon et al. used phylogenetic evaluation to reconstruct HIV spread within patients who had provided blood samples during life, and autopsy specimens through the “Last Gift” program [227,236]. Using full-length *env* sequences, they demonstrated that HIV persists by repopulation from multiple anatomical sources. When ART was stopped, large clonal populations emerged in blood that repopulated tissues throughout the body and viral exchanges occurred within brain areas and across the blood–brain barrier [227]. Analytic treatment interruption studies that compare CSF sequences before and after treatment interruption would further elucidate the behavior of the CNS reservoir.

The production of viral proteins in the CNS, even at very low levels, can induce inflammation or neural toxicity, which may manifest as neurocognitive deficits [237]. Fully suppressive ART has not been able to eliminate the neuropathogenic effects of HIV in the CNS [238]. Evidence of ongoing inflammation, immune activation, and neuronal injury have been detected in long-term well-controlled patients [238]. Moreover, imaging studies have shown persistent brain inflammation in ART-suppressed patients. These findings are likely related to the production of replication-incompetent virus or viral proteins from defective proviruses.

The HIV Tat protein has been reported to contribute significantly to the CNS effects of HIV [239,240,241]. Tat is present in the CNS even during suppressive ART [241]. In the CNS, Tat interacts with diverse cells, including microglia, astrocytes, microvascular endothelial cells, and neurons to dysregulate gene expression and engender cellular activation, inflammation, neurotoxicity, and structural damage [240]. Specific effects may be dependent on the HIV subtype [240]. A study of 68 HIV-positive individuals on ART, and 25 HIV-negative controls, showed that CSF Tat concentrations may actually increase after the initiation of ART, indicating that it is not inhibited by targeted therapy. Over half of the Tat found in the CNS was demonstrated to be functional on the basis of the retention of the transactivation activity. These findings suggest that Tat may be a quantifiable marker of proviral populations in the CNS [239]. Another study characterized 44 unique *tat* alleles from the brain, CSF, and spinal cord, and blood/lymphoid tissue HIV isolates from five subjects with HIV-associated dementia. Consistent tissue-specific compartmentalization was not detected. However, the segregation of CNS and non-CNS *tat* alleles was noted; alterations were predominantly in the transactivation domain [242]. This indicates that the CNS contains distinct proviral sequences, which may be expressed even when the provirus is not replication-competent and is consistent with unique CNS clones. Findings of identical Env sequences in the CSF after years of suppressive ART [231], and identical sequences in multiple compartments, including the brain, by single-genome sequencing also support the role of clonal expansion in the CNS.

Thus, the CNS does serve as an HIV reservoir, playing a critical role in persistence, the clinical manifestations of HIV, and viral repopulation when effective ART is no longer present. Furthermore, sequence analyses show that the CNS may contain clones or provirus that are distinct from those in other compartments.

Lymphatic tissues also contribute to persistent viremia, most likely because of low-level release from existing clonally expanded populations rather than cycles of new infection. It has been suggested that ongoing production from lymphoid tissue sanctuary sites, where drug concentrations may be inadequate, replenishes circulating virus that can traffic to blood or other lymphoid tissues [108]. Subsequent work has not shown evidence of ongoing replication cycles in lymphoid tissues [3,243]. The production of the virus and clonal expansion has been demonstrated in HIV controllers who have natural virologic control. In one study of natural controllers, viruses with the genetic and transcriptional attributes of active replication were identified in lymph nodes [244]. In blood, inducible proviruses of archival origin were detected among clonally expanded cells. Common to both blood and lymph nodes was a small population of circulating CD4 T cells found to contain inducible provirus of recent origin. Hence, HIV replication in lymphoid tissue, the clonal expansion of infected cells, and the recirculation of recently infected cells maintain the virus in the setting of an effective antiviral immune response.

The gastrointestinal tract is another important source of HIV replication, serving as a major source of the virus early in the disease course. Gut-associated lymphatic tissues (GALT) can be infected very early in acute HIV infection and can suffer a massive depletion of CD4 T cells in as little as 4 weeks [245]. The immunologic and structural disruption of the gut mucosa occurs rapidly after infection [246]. With ART, CD4 T-cell populations may be restored in the GI tract. This is a slow process related to polyfunctional anti-HIV cellular responses as well as Th17+ CD4 T cell accumulation [247]. Remaining gut cells with proviral DNA serve as a viral reservoir and a source of persistent low-level viremia. However, the GI tract does not seem to be an isolated anatomic sanctuary, given the lack of demonstrated compartmentalization between the gut and peripheral blood [248]. Highly sensitive techniques have been developed to measure gut viral reservoirs, which involve purifying lamina propria leukocytes from gut biopsies, followed by ddPCR to quantify HIV DNA [249].

Another important HIV reservoir is the genital tract. It is often the primary site of infection for transmission events via sexual contact and virus from the genital tract may be transmitted to others. The uterine cervix may be an important reservoir, for studies have identified instances in which rebound virus with ART cessation is genetically identical to cervical sequences [250]. Some studies have found that HIV-1 populations in cervical and vaginal tissue may have genetic features that differ from plasma virus, suggesting some degree of compartmentalization [251,252,253]. Compartmentalization and clonal expansion in semen samples have also been identified [254]. Plasma HIV RNA is an important predictor in genital HIV-1 shedding [255].

The bone marrow has been reported to be a potential anatomic reservoir. While hematopoietic stem and progenitor cells (CD34+ cells) are long-lived cells and are susceptible to HIV infection according to in vitro studies [256,257], provirus has not been conclusively demonstrated in stem cells in vivo. Some in vivo studies have detected the susceptibility of these cells to HIV infection, particularly those with high expression of CD4, while others have failed to identify HIV-1 DNA in purified CD34+ populations [258,259]. HIV DNA has been detected in other bone marrow populations, with more differentiated cells demonstrating the greatest susceptibility [260]. Furthermore, HIV *env*-specific PET-MR imaging has revealed higher activity in the bone marrow of ART-suppressed HIV-infected patients compared to uninfected controls [261]. The viability of bone marrow as an HIV reservoir is critically important because progenitor cells are not only long-lived, but could also give rise to many HIV-infected progeny, which would need to be addressed for successful HIV eradication.

**Figure 6:**
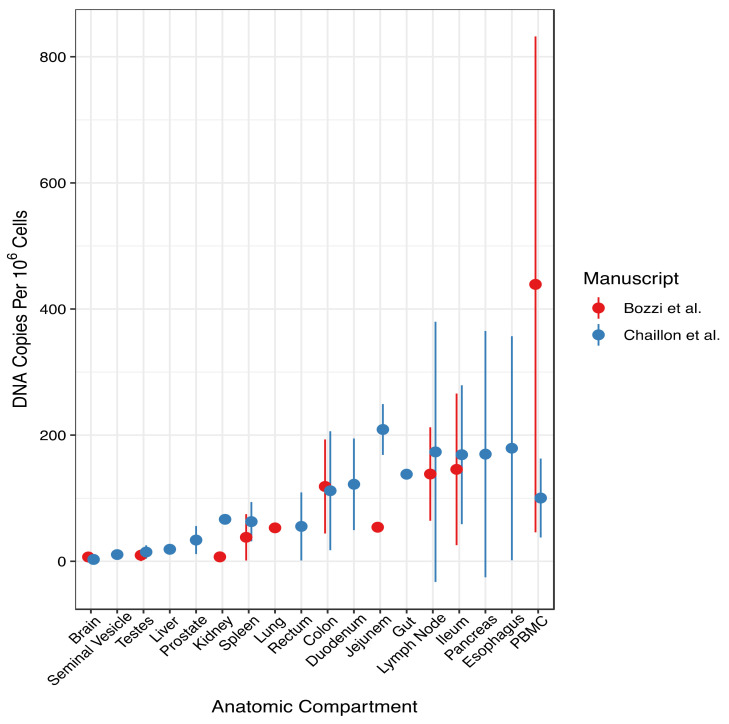
HIV DNA by Anatomic Compartment. This figure depicts copies of HIV DNA-per-million cells quantified by ddPCR at various anatomic sites, as described by two research groups. HIV DNA serves as an estimate of the number of proviruses present. A total of 10 individuals are represented in this figure, 8 of whom had undetectable plasma viral RNA until death and 2 of whom discontinued ART in the days or weeks before death. Each dot represents mean HIV DNA copies across all participants in that study at each anatomic site, with bars that extend to one SD above and below the mean value. Dots that do not have SD bars represent data from one individual, and thus SD could not be calculated. Data are from Bozzi et al. [3] and Chaillon [227].

HIV-infected cells have been documented in a variety of tissues in addition to the ones discussed above. Further studies on the viral dynamics in specific locations will be critical for developing effective cure strategies. The quantification of HIV DNA copies per million cells across many anatomic compartments from two studies is represented in Figure 6.

## 7. Implications of Persistence for Cure Strategies

HIV eradication has been reported (see below) but faces a number of obstacles, including low-level viral persistence during ART, the long-term persistence of HIV-infected cells, and the latent infection of resting CD4 T cells. Broadly deployable cure approaches will need to address the clonal expansion of latently infected cells as it propagates the reservoir and provides a source for the resurgence of infection. While low-level viral production during ART and the long half-life of some HIV-infected cells present potential targets for interfering with persistence [262], clonal expansion is more difficult to detect and target.

Cure strategies aim to achieve viral remission without ART. A number of approaches have been proposed, including “shock and kill”, “block and lock”, antibody therapy, genome editing, and hematopoietic stem cell transplantation (HSCT). HSCT is the only method that has successfully achieved the eradication of HIV infection. While this provides insight into the mechanisms of cure and demonstrates that it is achievable, HSCT may itself be life-threatening and is not practical for broad deployment. The “shock and kill” and “block and lock” approaches have not been able to produce the sustained inhibition of replication but could, theoretically, be implemented on a larger scale. Ongoing studies continue to explore these and other potential cure strategies, such as gene editing and vaccination [263].

“Shock and kill” was one of the first latency-reversing investigational cure approaches, with detailed in vitro and in vivo studies. It seeks to eliminate the proviral reservoir via latency-reversing agents (LRA) that unmask infected cells (“shock”) via the induction of viral protein production, thereby facilitating targeting by immune responses or viral cytopathic effectors, such as HIV-specific CD8 cells (“kill”) [264]. Approaches to latency reversal include chromatin modulation by the inhibition of histone deacetylase or acetyltransferase, transcription activators, the unblocking of transcriptional elongation, immune checkpoint inhibitors, and post-transcriptional modification [265]. Synergistic mechanisms recently demonstrated in preclinical studies suggest that a mix of immune interventions may be required to both suppress factors that stabilize latency and activate those that disrupt latency [266,267]. Proapoptotic compounds, such as second mitochondria-derived activator of caspase (SMAC) mimetics and inhibitors of the regulator protein B cell lymphoma 2 (Bcl2) and the PI3K/Akt pathway, have also garnered interest [13].

Though in vitro studies have demonstrated clear evidence of latency reactivation, LRAs have not resulted in long term changes in the markers of reservoir size in vivo, and it is not known why agents that effectively reverse latency in vitro have not had success in vivo [10,268]. It is unclear how much of the observed increases in viral RNA are attributable to cells with replication-competent HIV and how viral evolution might be affected [269]. Furthermore, LRAs may impact the functions of effector and target cells, including CD8 and CD4 T cells, and thus influence the probability of killing or elimination [270]. They also have potential off-target effects that may limit their efficacy, such as T-cell toxicity, as well as clinical side effects, including CNS and liver toxicity [271,272]. Some LRAs actually promote survival. For example, histone deacetylase inhibitors, such as suberanilohydroxamic acid (SAHA), romidepsin, and panobinostat, actually suppress the ability of CTL to kill HIV-infected cells [273]. The induction of latent cell death by apoptosis and the inhibition of critical cell survival pathways, which are often hijacked by HIV proteins, may address this issue. For example, cancer chemotherapeutics that induce apoptosis might be combined with latency-reversing agents to eliminate infection [274]. Moreover, the work on latency reversal has been conducted primarily with subtype B. It will be important for factors influencing latency to be evaluated across global subtypes in order to ensure broad applicability [275].

In contrast to the “shock-and-kill” strategy that aims to eradicate the entire reservoir, the “block-and-lock” approach seeks to permanently silence all proviruses by mimicking natural latency. The ancient human endogenous retroviruses, which comprise approximately 8% of the human genome, are typically epigenetically silenced. Although human endogenous retrovirus sequences can be expressed in response to environmental factors or infection, their silencing mechanism provides a prototype for “block-and-lock” [276]. HIV silencing might be achieved by targeting different components of the transcription machinery. Hence, multiple mechanisms, including the sequestration of proteins required for transcription in an inactive state, can be employed to inhibit proviral transcription. “Block-and-lock” blocks HIV transcription using latency-promoting agents (LPAs) and locks the HIV promoter in a deep latent state via epigenetic modifications. Permanent control of the HIV promoter circumvents the need for ART. This, in combination with the high specificity of the sequences targeted, makes “block-and-lock” an attractive possibility for achieving ART free remission [13,277].

Multiple “block-and-lock” approaches are under investigation. The most advanced uses the Tat protein inhibitor, didehydro-cortistatin A, to silence transcription. Another employs 2-(quinolin-3-yl) acetic acid derivatives (LEDGINs), which inhibit the interaction of HIV integrase and the chromatin tethering factor, LEDGF/p75, to inhibit HIV integration [278]. DNA that does manage to integrate does so at sites less susceptible to reactivation. The applicability of LEDGINs beyond the initial infection phase is questionable given their target [279]. The inhibition of “facilitates chromatin transcription complex” (FACT), a histone chaperone that promotes transcription by destabilizing the nucleosomal structure, also promotes HIV latency. This has been demonstrated using curaxin, an antineoplastic agent that sequesters FACT [280,281]. RNA-induced epigenetic silencing is an alternative approach that utilizes short interfering or short hairpin RNA to maintain heterochromatin repression at the HIV 5′ LTR promoter. Heat shock protein 90, a chaperone protein required for folding and stabilizing other proteins, can be inhibited to suppress HIV transcription and replication [282]. The Jak-STAT pathway is integral to memory T-cell homeostasis. Ruxolitinib and tofacitinib, both Jak-STAT inhibitors that are FDA-approved for other applications, have been shown to block reactivation in primary CD4 T cells [283]. Additional block-and-lock agents under investigation include BRD4 modulators, mTOR inhibitors, kinase inhibitors, and triptolide.

Other cure strategies have focused on manipulating the host immune system to eradicate the virus. For example, type I interferons have antiviral activity [284], though their impact on the HIV reservoir is unclear. The question of whether boosting type I interferon signaling during acute infection results in a smaller reservoir or slows disease progression is being explored [285]. Some studies suggest that pegylated interferon-alpha treatment could reduce reservoir size, decrease HIV-1 integration, and increase NK cell cytotoxicity [286,287]. Moreover, pegylated interferon alpha 2b treatment plus ART has been shown, in a cohort of 20 patients, to decrease blood and GALT immune activation, reduce HIV-1 RNA+ cells in GALT, and decrease activated natural killer cells and macrophages [288]. This therapy might be useful in combination with other cure strategies [289]. Broadly neutralizing antibodies (bNAbs) have also been considered as an immunotherapy to treat or cure HIV. Such therapies involve the infusion of antibodies that can bind to and kill HIV-infected cells or prevent the infection of new cells [290,291]. Therapeutic vaccination to trigger the production of bNAbs is one potential application for cure. The emergence of resistance with rebound viremia, a lack of efficacy in preventing cell-to-cell viral transmission, and the uncertain effects on reservoir size have limited the efficacy of bNAbs as a treatment and cure strategy [291].

Genome-editing strategies are also being explored as a mechanism for achieving HIV cure without the need for ART [292]. Clustered and regularly interspaced short palindromic repeats (CRISPR-Cas9) and transcription activator-like effector nucleases (TALEN) specific for the CCR5 gene have been harnessed in an attempt to effect functional cure by preventing HIV from entering target cells [293]. Zinc finger nucleases (ZFN) have been evaluated for their ability to modify CCR5 expressing CD4 T cells, which were then infused during analytic treatment interruption. These ZFN-edited cells were found to augment the HIV-specific immune responses [294]. As gene-editing strategies progress in development, the prevention and mitigation of off-target effects will be essential [295].

In general, optimal cure strategies involve short-term interventions, so as not to become an alternative form of a long-term treatment to ART. Transient pharmacologic treatment may serve as an effective curative strategy in “shock-and-kill” approaches that use latency reversal followed by the elimination of infected cells by the immune system or another mechanism. However, short-term transient pharmacologic treatment, such as with inhibitors or activators, may not be sufficient for complete eradication approaches, including “block-and-lock”, because it may not achieve a durable effect. Similarly, the inhibition of a cellular enzyme would only persist while the inhibitor is present in adequate concentrations. Furthermore, the inhibitors of cellular enzymes that permanently affect an enzyme could affect other cellular functions, and an analysis of the off-target effects of these interventions will be a useful component of their evaluation.

HSCT is the only successful cure strategy thus far. It has relied on using donor cells that are homozygous for a 32-base-pair deletion in the chemokine coreceptor, CCR5 (CCR5∆32), which is required for infection by R5 viruses [296,297,298]. To end the cycle of new infection, the HIV-infected recipient must only have the R5 virus; viruses that rely on other receptors can still infect new cells and replicate. HSCT could, theoretically, be conducted with other infection-resistant cells to effect cure, but this has not yet been performed. Homozygously resistant cells would most likely be required as the heterozygosity of the ∆32 mutation has not been shown to prevent the progression of infection [297].

The “Berlin patient” was the first case of homozygous CCR5∆32 HSCT that effected a cure. This individual had acute myeloid leukemia and R5 HIV infection. After one transplant, the patient did not demonstrate viral rebound, despite discontinuing ART, and required a second transplant with aggressive conditioning to treat his neoplasm, suggesting that, after conditioning and transplant, the abundance of tumor cells was greater than the HIV-infected reservoir cells. The patient remained without viral rebound until his death more than a decade later [299,300]. The recurrence of the leukemia led to his death, and it is possible that the reservoir for the neoplasm remained more long-lived than that of the HIV infection.

A second example of HIV cure reported by Gupta and coworkers is the “London patient”. This individual was HIV-infected and had Hodgkin’s lymphoma. Like the “Berlin patient”, the “London patient” also underwent CCR5∆32 HSCT. Unlike the “Berlin patient,” the “London patient” did not have total body irradiation. After stopping ART, the “London patient” has maintained remission for at least 3 years with RNA undetectable at <1 copy/mL, RNA the below limits of detection from multiple anatomic locations, and viral outgrowth assays showing no recovery of HIV [301,302].

For both the London and Berlin patients, the HIV-specific antibody levels and avidities fell. The reason for the success of these transplants is not entirely certain. In addition to homozygosity for CCR5∆32, the graft recognition of HIV epitopes may have facilitated the elimination of HIV-infected cells. However, many other individuals living with HIV have undergone HSCT and have not been cured. For example, in non-CCR5∆32 HSCT, the persistence of HIV-infected cells in some reservoirs, despite cytotoxic therapy, allows these cells to transmit HIV to the graft when ART is interrupted. Cytotoxic therapy likely does not eradicate all HIV-infected cells, given that this therapy only targets actively dividing cells. While the Berlin and London cases do demonstrate that cure is possible, HSCT is not scalable and is itself associated with significant morbidity and mortality. It is possible that the risk can be lessened with reduced intensity conditioning and the omission of radiation. Additionally, there is a risk of the emergence of more cytotoxic X4 viruses with CCR5∆32 homozygous HSCT, as X4 viruses would still be able to infect new cells and replicate. The loss of CCR5 might also have off-target effects, such as the inhibition of immune responses to other pathogens, e.g., West Nile Virus. The ethics of using life-threatening eradication approaches, which have a risk of failure, for an HIV infection with which patients can live long lives with appropriate treatment, are questionable.

Cure strategies will need to be optimized and likely combined to achieve robust efficacy. Using “block-and-lock” or “shock-and-kill” with stem cell methods has been proposed as a means to achieve cure while minimizing toxicity. Optimization and the adaptation of HSCT with resistant donor cells also provides promise for a select group of PLWH that is already at an increased risk because of comorbidities. For all eradication approaches, the latent reservoir and its maintenance by clonal expansion will need to be addressed. It is clear that proviruses from clonal populations can drive the resurgence of infection once ART is stopped. Strategies that halt clonal expansion and eliminate the reservoir, or that prevent new infection even when the virus is circulating, will be needed.

The elimination of infected cells will need to take place in different anatomic compartments, including the lymphoid tissues, CNS, genital tract, and other locations with distinct characteristics. Eradication strategies that are effective in the peripheral blood may not be equally useful in all compartments or may be impacted by the local milieu. Moreover, proviruses that can propagate in a specific compartment may be differentially susceptible to interventions. The evaluation of cure strategies must assess the residual provirus in less accessible compartments. This is particularly important in the CNS, which is protected by the blood–brain barrier and where direct and indirect cytotoxicity can result from the presence of viral proteins as well as ongoing replication. Given the mechanisms of clonal expansion, even a small amount of provirus can renew reservoirs without being detected and, hence, support the resurgence of infection when the suppressive forces abate. Curative strategies will require flexibility for efficacy in diverse anatomic settings, particularly so for CNS HIV populations. CNS populations may be quite distinct [233,234]. The provirus that gives rise to the sequences detected in the CNS may reside in multiple cell lineages, including perivascular macrophages, microglia, and astrocytes. It remains unclear if CNS clones will be differentially susceptible to scalable cure strategies in development [303].

The clinical studies of several patient groups, in addition to individuals who have undergone transplant, shed light on our understanding of HIV persistence, including the natural control of HIV infection in elite controllers, post-treatment controllers, and rare examples of individuals with extreme control and seroreversion. For example, an examination of post-treatment controllers is essential for understanding what differentiates a successful immunologic response to HIV from one that fails. The characterization of such cohorts may identify strategies to achieve virologic control during the cessation of ART via the stimulation of efficacious responses from the host immune system. Although it is beyond the scope of this review, an analysis of these patients will yield new insights into the requirements for HIV control [178,304,305,306,307]. 

Advancing the cure agenda requires a better understanding of the role of clonal expansion, and a number of critical questions remain unanswered:

1. What is the role of clonal expansion in maintaining a stable level of HIV-infected cells during prolonged ART?
2. Do interventions designed to reduce the HIV reservoir affect the clonal expansion of HIV-infected cells? Does latency reversal lead to the clonal expansion of infected cells? Does block-and-lock result in a loss of clones?
3. How does the HIV-infected cell population respond to cytotoxic chemotherapy? Understanding whether HIV-infected cells are preferentially lost or whether clonal expansion results in repopulation post-chemotherapy will provide new insights regarding eradication requirements;
4. Are clones widely distributed in anatomic compartments? Does clonal expansion occur in all tissues?
5. Are there unique strategies to target clonal expansion? For which cure approaches would this be necessary?

## 8. Conclusions

The clonal expansion of HIV-infected cells plays a central role in the maintenance of the HIV reservoir. Notably, host immune mechanisms are largely responsible for HIV persistence, including antigen-driven expansion, homeostatic proliferation, and generalized immune activation. Provirus-driven expansion can also occur and is responsible for the persistence of a minority of clones. Clonal expansion presents a barrier to current cure approaches other than HSCT. Addressing clonal expansion will likely be necessary for an effective scalable cure. Cure approaches that prevent the expression of, or that directly identify and eliminate proviral sequences in clones, should undergo further development. A specific evaluation of the cure approaches on clonal populations will be essential to determining the likelihoods of success of these strategies.

Key Points:

1. The principal mechanism of HIV persistence is long-lived immune cells that undergo clonal expansion. This process is common and includes the expansion of HIV-infected cells;
2. ARV intensification and phylogenetic studies suggest that the infection of new cells is rare during ART;
3. Both replication-competent and defective proviruses undergo clonal expansion and contribute to morbidity in PLWH;
4. There are four potential mechanisms of the clonal expansion of HIV-infected CD4 T cells. Integration may play a role in some instances of clonal expansion and its inhibition alters HIV decay dynamics;
5. Clones come to dominate the proviral landscape on ART, but individual clones wax and wane over time.
6. Successful HIV cure strategies will need to overcome the effects of clonal expansion and target the anatomic sites in which latently infected cells persist.

## Figures and Tables

**Table 1:** Integrase strand transfer inhibitors.

INSTI—1st Generation	Half-Life (T_1/2_)	Major Resistance Mutations		Less Common Resistance Mutations
		Shared	Specific	
Raltegravir	~9 h	T66K, E92Q, G118R, E138KAT, G140SAC, Q148HRK, N155H, R263K	T66AI, Y143RCH	(H51Y), (L74M), (T97A), F121Y, Y143KSGA, V151L, N155ST
Elvitegravir	~13 h		T66AI, S147G	H51Y, E92VG, F121Y, P145S, Q146P, Q148N, V151LA, S153YF, N155ST
**INSTI—2nd generation**				
Dolutegravir	~14 h			(H51Y), (L74MF), (T97A), (G149A), V151L, S153YF, S230R, (M50I)
Bictegravir	~17 h			(H51Y), S153YF
**INSTI—Long acting**				
Cabotegravir (injection)	~40 days [86]	In vitro: E138K, Q148K, G140S Clinical trials: G118R, E138K, G140SA, Q148KR, N155H		In vitro: T124A, Q146L, S153Y, I162M, (C56S, V72I, L74M, V75A, T122N, G149A, M154I) Clinical trials: G140R, (T66K, L74M, E92Q. E138A, Y143H)

Adapted from the Guidelines for the Use of Antiretroviral Agents in Adults and Adolescents Living with HIV, Appendix B, Table 6 (https://clinicalinfo.hiv.gov/en/guidelines/adult-and-adolescent-arv/characteristics-integrase-inhibitors?view=full accessed on 29 November 2021), the Stanford University HIV Drug Resistance Database (https://hivdb.stanford.edu/dr-summary/resistance-notes/INSTI/ accessed on 29 November 2021), and Cabenuva prescribing information (https://gskpro.com/content/dam/global/hcpportal/en_US/Prescribing_Information/Cabenuva/pdf/CABENUVA-PI-PIL-IFU2-IFU3.PDF, accessed on 29 November 2021). Mutation nomenclature shows the consensus wildtype amino acid abbreviation, position in the HIV-1 genome, and the mutated amino acid abbreviation. Mutations in parenthesis indicate mutations that do not alone induce resistance.

**Table 2:** ARV intensification studies and perspectives.

Year	Authors	Title	Significance
2020	López-Huertas, M.R.; Gutiérrez, C.; Madrid-Elena, N.; Hernández-Novoa, B.; Olalla-Sierra, J.; Plana, M.; Delgado, R.; Rubio, R.; Muñoz-Fernández, M.Á.; Moreno, S. [134]	Prolonged administration of maraviroc reactivates latent HIV in vivo, but it does not prevent ARV-free viral rebound	MVC intensification for 48 weeks increased residual viremia and episomal 2-LTR DNA circles suggesting that maraviroc could reactivate latent HIV. MVC induced an increase in cell-associated HIV RNA. Rapid rebound of viremia occurred after ART discontinuation. MVC can reactivate latent HIV in vivo but does not reduce the reservoir.
2018	Kityo, C.; Szubert, A.J.; Siika, A.; Heyderman, R.; Bwakura-Dangarembizi, M.; Lugemwa, A.; Mwaringa, S.; Griffiths, A.; Nkanya, I.; Kabahenda, S.; Wachira, S.; Musoro, G.; Rajapakse, C.; Etyang, T.; Abach, J.; Spyer, M.J.; Wavamunno, P.; Nyondo-Mipando, L.; Chidziva, E.; Nathoo, K.; Klein, N.; Hakim, J.; Gibb, D.M.; Walker, A.S.; Pett, S.L.; REALITY trial team [135]	Raltegravir-intensified initial antiretroviral therapy in advanced HIV disease in Africa: A randomized controlled trial	12 weeks of RAL intensification reduced HIV viremia significantly faster than standard triple-drug ART but did not reduce mortality or clinical events. There was no excess of IRIS-compatible events, suggesting that ISNTIs are safe in severe immunocompromise.
2018	Chaillon, A.; Gianella, S.; Lada, S.M.; Perez-Santiago, J.; Jordan, P.; Ignacio, C.; Karris, M.; Richman, D.D.; Mehta, S.R.; Little, S.J.; Wertheim, J.O.; Smith, D.M. [136]	Size, composition, and evolution of HIV DNA populations during early antiretroviral therapy and intensification with maraviroc	Low-level viremia persisted when standard ART +/− maraviroc intensification was started during acute infection. MVC did not impact viral evolution, diversity, or population structure over 90 weeks. This does not support propagation of infection as a source of viremia on ART.
2018	Henrich, T.J. [137]	Dolutegravir intensification and HIV persistence: 3 + 1 = 3	There is little clinical support for intensifying existing regimens with additional drug classes. Three-drug ART remains the mainstay of therapy unless resistance requires otherwise.
2018	Martinez-Picado, J.; Zurakowski, R.; Buzon, M.J.; Stevenson, M. [133]	Episomal HIV-1 DNA and its relationship to other markers of HIV-1 persistence	A steady state of de novo infection in ART-suppressed individuals may drive immune activation and inflammation, reflecting residual viral reservoir activity during effective ART.
2018	Puertas, M.C.; Gómez-Mora, E.; Santos, J.R.; Molto, J.; Urrea, V.; Moron-Lopez, S.; Hernandez-Rodriguez, A.; Marfil, S.; Martínez-Bonet, M.; Matas, L.; Muñoz-Fernández, M.A.; Clotet, B.; Blanco, J.; Martinez-Picado, J. [138]	Impact of intensification with raltegravir on HIV-1-infected individuals receiving monotherapy with boosted PIs	ART intensification with 24 weeks of RAL, in patients receiving maintenance monotherapy with ritonavir-boosted DRV or LPV, transiently increased 2-LTR circles and did not change the proportion of patients with detectable residual viremia.
2018	Rasmussen, T.A.; McMahon, J.; Chang, J.J.; Audsley, J.; Rhodes, A.; Tennakoon, S.; Dantanarayana, A.; Spelman, T.; Schmidt, T.; Kent, S.J.; Morcilla, V.; Palmer, S.; Elliott, J.H.; Lewin, S.R. [11]	The effect of antiretroviral intensification with dolutegravir on residual virus replication in HIV-infected individuals: a randomized placebo-controlled double-blind trial	A total of 8 weeks of ART intensification with dolutegravir did not change 2-LTR circles. Given that the inhibition of active replication would increase 2-LTR circles, residual replication was not demonstrated.
2017	Kim, C.J.; Rousseau, R.; Huibner, S.; Kovacs, C.; Benko, E.; Shahabi, K.; Kandel, G.; Ostrowski, M.; Kaul, R. [139]	Impact of intensified antiretroviral therapy during early HIV infection on gut immunology and inflammatory blood biomarkers	Intensification of FTC/TDF + LPV/r, started in early HIV infection with RAL + MVC vs. placebo, did not result in differences in blood or gut immune parameters. Most parameters improved, but did not completely normalize, in both groups.
2017	Wang, X.; Mink, G.; Lin, D.; Song, X.; Rong, L. [140]	Influence of raltegravir intensification on viral load and 2-LTR dynamics in HIV patients on suppressive antiretroviral therapy	On the basis of multi-stage models, RAL intensification has a minor effect on viral load and 2-LTR in HIV patients on suppressive ART.
2014	Lafeuillade, A.; Assi, A.; Poggi, C.; Bresson-Cuquemelle, C.; Jullian, E.; Tamalet, C. [141]	Failure of combined antiretroviral therapy intensification with maraviroc and raltegravir in chronically HIV-1-infected patients to reduce the viral reservoir: the IntensHIV randomized trial	Intensification of protease-inhibitor-based ART with MVC and RAL does not impact blood proviral DNA but can decrease cell-associated HIV RNA and CD8 activation.
2014	Puertas, M.C.; Massanella, M.; Llibre, J.M.; Ballestero, M.; Buzon, M.J.; Ouchi, D.; Esteve, A.; Boix, J.; Manzardo, C.; Miró, J.M.; Gatell, J.M.; Clotet, B.; Blanco, J.; Martinez-Picado, J.; MaraviBoost Collaborative Group [142]	Intensification of a raltegravir-based regimen with maraviroc in early HIV-1 infection	Addition of MVC to TDF / FTC + RAL, started in early HIV-1 infection, results in modest reduction in reservoir size at 48 weeks. Plasma viremia decreased in both groups but remained detectable in several subjects.
2013	Gutiérrez, C.; Hernández-Novoa, B.; Vallejo, A.; Serrano-Villar, S.; Abad-Fernández, M.; Madrid, N.; Díaz, L.; Moreno, A.; Dronda, F.; Zamora, J.; Muñoz-Fernández, M.A.; Moreno, S. [143]	Dynamics of the HIV-1 latent reservoir after discontinuation of the intensification of antiretroviral treatment: results of two clinical trials	A total of 48 weeks of ART intensification, followed by a return to baseline ART for 24 weeks, found fewer latently infected memory CD4 T cells with replication-competent virus and 2-LTR circles. This suggests intensification has persistent effects after discontinuation but does not eliminate the reservoir.
2013	Negredo, E.; Massanella, M.; Puertas, M.C.; Buzon, M.J.; Puig, J.; Pérez-Alvárez, N.; Pérez-Santiago, J.; Bonjoch, A.; Moltó, J.; Jou, A.; Echeverría, P.; Llibre, J.M.; Martínez-Picado, J.; Clotet, B.; Blanco, J. [144]	Early but limited effects of raltegravir intensification on CD4 T-cell reconstitution in HIV-infected patients with an immunodiscordant response to antiretroviral therapy	RAL intensification in immunodiscordant patients (CD4 < 350 on suppressive ART) did not change proviral DNA levels; episomal DNA and ultrasensitive plasma viral load were barely detected; CD4 T-cell increases were limited. Residual viral replication is not the main cause of poor CD4 T-cell recovery in immunodiscordance.
2013	Sharkey, M. [145]	Tracking episomal HIV DNA: implications for viral persistence and eradication of HIV	The 2-LTR circles are surrogates for replication that can be used to monitor the effects of ART intensification, the sources of viral rebound, and viral variants contributing to treatment failure.
2012	Chege, D.; Kovacs, C.; La Porte, C.; Ostrowski, M.; Raboud, J.; Su, D.; Kandel, G.; Brunetta, J.; Kim, C.J.; Sheth, P.M.; Kaul, R.; Loutfy, M.R. [146]	Effect of raltegravir intensification on HIV proviral DNA in the blood and gut mucosa of men on long-term therapy: a randomized controlled trial	Intensification of suppressive ART with RAL was not associated with differences in blood or gut HIV proviral levels or CD4 T-cell increases in this double-blind randomized placebo-controlled study.
2012	Hatano, H.; Scherzer, R.; Wu, Y.; Harvill, K.; Maka, K.; Hoh, R.; Sinclair, E.; Palmer, S.; Martin, J.N.; Busch, M.P.; Deeks, S.G.; Hsue, P.Y. [147]	A randomized controlled trial assessing the effects of raltegravir intensification on endothelial function in treated HIV infection	Addition of RAL to suppressive ART did not affect the rate of change of the flow-mediated vasodilation of the brachial artery, a marker of endothelial function.
2012	Llibre, J.M.; Buzón, M.J.; Massanella, M.; Esteve, A.; Dahl, V.; Puertas, M.C.; Domingo, P.; Gatell, J.M.; Larrouse, M.; Gutierrez, M.; Palmer, S.; Stevenson, M.; Blanco, J.; Martinez-Picado, J.; Clotet, B. [148]	Treatment intensification with raltegravir in subjects with sustained HIV-1 viremia suppression: a randomized 48-week study	This prospective open-label randomized study of 48 weeks of RAL intensification in patients on suppressive ART did not show a change in total or integrated HIV DNA. Ultrasensitive VL remained stable.
2011	Buzon, M.J.; Codoñer, F.M.; Frost, S.D.W.; Pou, C.; Puertas, M.C.; Massanella, M.; Dalmau, J.; Llibre, J.M.; Stevenson, M.; Blanco, J.; Clotet, B.; Paredes, R.; Martinez-Picado, J. [149]	Deep molecular characterization of HIV-1 dynamics under suppressive HAART	RAL intensification of suppressive ART resulted in a transient increase in episomal DNA in most subjects. In subjects with episomal DNA increases, immune activation was higher at baseline and normalized with RAL intensification, suggesting that active replication may persist and drive immune activation.
2011	Dahl, V.; Lee, E.; Peterson, J.; Spudich, S.S.; Leppla, I.; Sinclair, E.; Fuchs, D.; Palmer, S.; Price, R.W. [150]	Raltegravir treatment intensification does not alter cerebrospinal fluid HIV-1 infection or immunoactivation in subjects on suppressive therapy	RAL intensification of suppressive ART did not reduce intrathecal immunoactivation or alter CSF viral load. Patients had very low CSF viral loads, regardless of intensification.
2010	Archin, N.M.; Cheema, M.; Parker, D.; Wiegand, A.; Bosch, R.J.; Coffin, J.M.; Eron, J.; Cohen, M.; Margolis, D.M. [151]	Antiretroviral intensification and valproic acid lack sustained effect on residual HIV-1 viremia or resting CD4 T-cell infection	Addition of valproic acid (HDAC inhibitor) and RAL, valproic acid and T20 or T20, failed to progressively reduce the frequency of resting CD4 T-cell infection or ablate low-level viremia.
2010	Buzon, M.J.; Massanella, M.; Llibre, J.M.; Esteve, A.; Dahl, V.; Puertas, M.C.; Gatell, J.M.; Domingo, P.; Paredes, R.; Sharkey, M.; Palmer, S.; Stevenson, M.; Clotet, B.; Blanco, J.; Martinez-Picado, J. [131]	HIV-1 replication and immune dynamics are affected by raltegravir intensification of HAART-suppressed subjects	RAL intensification of suppressive ART resulted in a transient increase in episomal DNA and normalization of immune activation. This suggests that replication persists in some infected individuals on ART and drives immune activation.
2010	Hammer, S.M.; Ribaudo, H.; Bassett, R.; Mellors, J.W.; Demeter, L.M.; Coombs, R.W.; Currier, J.; Morse, G.D.; Gerber, J.G.; Martinez, A.I.; Spreen, W.; Fischl, M.A.; Squires, K.E.; AIDS Clinical Trials Group (ACTG) 372A Study Team [1]	A randomized placebo-controlled trial of abacavir intensification in HIV-1-infected adults with virologic suppression on a protease-inhibitor-containing regimen	ABC intensification of IDV + AZT + 3TC in patients with plasma viral loads <500 copies/mL did not confer clinical or virologic benefit. Proportion of subjects with plasma viral loads < 50 copies/mL, CD4 T-cell increase, rates of intermittent viremia, suppression of plasma viral loads < 6 copies/mL, and HIV proviral DNA in peripheral blood mononuclear cells (PBMC) were not different.
2010	McMahon, D.; Jones, J.; Wiegand, A.; Gange, S.; Kearney, M.; Palmer, S.; McNulty, S.; Metcalf, J.A.; Acosta, E.; Rehm, C.; Coffin, J.M.; Mellors, J.W.; Maldarelli, F. [152]	Short-course raltegravir intensification does not reduce persistent low-level viremia in patients with HIV-1 suppression during receipt of combination antiretroviral therapy	The 4 weeks of ART intensification with RAL did not decrease persistent viremia in subjects receiving suppressive ART. This indicates that rapidly cycling HIV-infected cells were not present.
2010	Yilmaz, A.; Verhofstede, C.; D’Avolio, A.; Watson, V.; Hagberg, L.; Fuchs, D.; Svennerholm, B.; Gisslén, M. [153]	Treatment intensification has no effect on the HIV-1 central nervous system infections in patients on suppressive antiretroviral therapy	Switch-over intensification with 4 weeks of MVC or LPV/r (good CNS penetration) and 4 weeks with T20 (poor CNS penetration) did not change residual CSF HIV RNA or intrathecal immunoactivation in patients on ART. This does not support ongoing viral replication in the CNS.
2009	Dinoso, J.B.; Kim, S.Y.; Wiegand, A.M.; Palmer, S.E.; Gange, S.; Cranmer, L.; O’Shea, A.; Callender, M.; Spivak, A.; Brennan, T.; Kearney, M.F.; Proschan, M.A.; Mican, J.M.; Rehm, C.A.; Coffin, J.M.; Mellors, J.W.; Siliciano, R.F.; Maldarelli, F. [154]	Treatment intensification does not reduce residual HIV-1 viremia in patients on highly active antiretroviral therapy	ART intensification with EFV, LPV/r, or ATV/r did not decrease viremia. Lack of response was not associated with the drug-resistant virus or suboptimal drug concentrations. Viremia likely due to output from stable reservoirs, not ongoing cycles of replication.
2003	Havlir, D.V.; Strain, M.C.; Clerici, M.; Ignacio, C.; Trabattoni, D.; Ferrante, P.; Wong, J.K. [155]	Productive infection maintains a dynamic steady state of residual viremia in people infected with human immunodeficiency virus type 1 treated with suppressive antiretroviral therapy for 5 years	Addition of ABC in patients suppressed on IDV and EFV decreased HIV viral load but residual viremia plateaued at 3.2 to 23 copies/mL. Residual viremia level was established by 9 months, predicted by baseline proviral DNA, and remained constant for 5 years.

3TC: lamivudine; ABC: abacavir; ATV: atazanavir; AZT: zidovudine; c: cobicistat; DRV: darunavir; FTC: emtricitabine; IDV: indinavir; LPV: lopinavir; MVC: maraviroc; r: ritonavir; RAL: raltegravir; T20: enfurvitide; TDF: tenofovir disoproxil fumarate.

**Table 3:** HIV receptors, coreceptors, and cofactors.

Cell Type	Ligands on HIV Envelope	Primary Receptor	Coreceptor	Cofactors	Special Considerations
**CD4+ T Cells**	ICAM-1, gp41, gp120	CD4	CCR5/CXCR4	*α*4*β*7 Integrin, CD26, LFA-1	Primary target for HIV cure.
**Macrophages**	MHC II, PS, ICAM-1, gp41, gp120	CD4	CCR5/CXCR4	Syndecans, integrins, alternate chemokine cytokine receptors *, Annexin A2, MMR, LFA-1	Present in key anatomic locations, such as lymph nodes, central nervous system (microglia), and gut [223,224].
**Antigen-Presenting Cells (e.g., dendritic cells, Langerhans cells)**	gp41, gp120	CD4 (productive infection); DC-SIGN, DCIR, Langerin (non-productive infection) **	CCR5/CXCR4 (for infections occurring via CD4 primary receptor)	N/A	There is some debate whether Langerhans cells are susceptible to productive HIV infection [212,214,215,225].

* alternate chemokine cytokine receptors include CCR1, CCR2b, CCR3, CCR8, CX3CR1, CXCR6, chemokine-binding protein 2 (CCBP2), G protein-coupled receptor 1 (GPR1), GPR15, formyl peptide receptor 1, and apelin receptor. ** nonproductive infection occurs via transinfection. CCR5: CC chemokine receptor type 5; CD4: cluster of differentiation 4; CXCR4: CXC chemokine receptor type 4; DC-SIGN: dendritic cell-specific ICAM 3-grabbing nonintegrin; DCIR: dendritic cell immunoreceptor; ICAM-1: intracellular adhesion molecule-1; LFA-1: lymphocyte function-associated antigen 1; MHC II: major histocompatibility complex II; MMR: macrophage mannose receptor; PS: phosphatidylserine.
